# Gold Plasmophores for Plasmon-Enhanced Photoelectrochemical Sensing Based on Metal Nanostructures

**DOI:** 10.1021/acs.jpca.6c01457

**Published:** 2026-07-24

**Authors:** Lizhen Chen, Robert R. Zierold, Miao Feng, Stefan Werner, Gero Göbel, Robert H. Blick, Wolfgang J. Parak, Fred Lisdat, Indranath Chakraborty

**Affiliations:** † Fachbereich Physik, Center for Hybrid Nanostructures (CHyN), 14915 Universität Hamburg, 22761 Hamburg, Germany; ‡ Fachbereich Chemie, Universität Hamburg, Hamburg 20146, Germany; § Biosystems Technology, Institute for Applied Life Sciences, Technical University Wildau, D-15745 Wildau, Germany; ∥ School of Nano Science and Technology, 30133 Indian Institute of Technology Kharagpur, Kharagpur 721302, India

## Abstract

Surface plasmon-enhanced photoelectrochemical (PE–PEC) processes have garnered growing interest due to their high sensitivity and reduced energy consumption. In this work, a semiconductor-free PE–PEC platform is developed by integrating plasmonic gold nanorods (Au NRs) with gold nanoclusters (Au NCs). The system is constructed by depositing a monolayer of Au NRs with a thin SiO_2_ spacer layer onto an Au NC-modified gold electrode. Experimental results supported by theoretical simulations demonstrate that near-field enhancement arising from plasmonic coupling between adjacent Au NRs significantly improves charge carrier excitation and photocurrent generation in the Au NCs. The PE–PEC platform was evaluated for the detection of biologically relevant model analytes, namely, hydrogen peroxide and catechol. Compared to electrodes modified with Au NCs alone, the PE–PEC system exhibits markedly enhanced sensitivity for both analytes. These results highlight the potential of rationally designed plasmon-enhanced architectures for improving photoelectrochemical performance and advancing multifunctional PEC-based bioanalysis.

## Graphical abstract

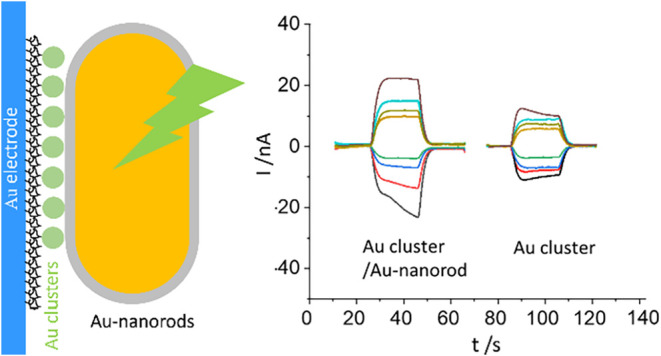

## 1 Introduction

Photoelectrochemical (PEC) analytical methods have emerged as powerful tools for biological sensing due to their high sensitivity, low background signals, and the synergistic combination of light excitation with electrochemical readout.^1–3^ Additional advantages, such as multiplexing capabilities, further enhance their analytical potential.^4,5^ Central to the PEC performance is the choice of photoactive material. While semiconductors-based systems, including quantum dots, offer favorable optoelectronic properties, concerns related to toxicity and material complexity limit their applicability in biological environments.^6^ Although strategies have been developed to overcome toxic effects,^7^ the search for biocompatible and low-toxic photoactive materials for efficient PEC performance and ease of production is an ongoing task.

Gold nanoclusters (Au NCs) represent a promising alternative owing to their molecular-scale dimensions, photostability, and semiconductive behavior. Au NCs have been successfully applied in PEC sensing schemes for hydrogen peroxide, oxygen, and in combination with enzyme reactions for glucose, sarcosine or environmental pollutants.^8–11^ For example, Xiao et al. reported the development of Au cluster-decorated, highly ordered nanoporous layer-covered TiO_2_ nanotube heterostructures (Au_*x*_/NP-TNTA) that significantly enhanced the photocatalytic oxidation of organic pollutants.^12^ However, their relatively weak visible-light absorption and limited charge separation efficiency restrict overall PEC performance, motivating the development of strategies to enhance their photoelectrochemical activity.^13,14^

One effective approach to improve PEC efficiency involves coupling photoactive materials with plasmonic nanostructures. Plasmonic metals such as gold and silver can amplify local electromagnetic fields via localized surface plasmon resonance (LSPR), thereby enhancing light absorption and charge carrier generation in adjacent material.^15–17^ Previous studies have demonstrated that integrating plasmonic nanoparticles with Au NCs or semiconductor systems can significantly increase photocurrent densities and sensing performance.^11,18,19^ Importantly, the magnitude of plasmonic enhancement strongly depends on the distance between plasmonic and photoactive components, with nanoscale separations enabling pronounced near-field coupling effects.^20,21^ For example, Mondes et al. have reported that the electric near-field enhancement resulting from the coupling between plasmonic nanoparticles with small interparticle distances, i.e., around 4 nm, is particularly pronounced.^22^ This experimental result has been accompanied by Finite-difference time-domain (FDTD) simulations that quantified the dependence of field enhancement on the interparticle distance. However, a direct contact between the plasmonic metal nanostructures with the semiconductor materials might reduce the enhancement effect due to a potential nonradiative energy transfer from the photoexcited semiconductor materials to the plasmonic metal nanostructures.^23,24^

Gold nanorods (Au NRs) are particularly attractive plasmonic enhancers due to their tunable longitudinal and transverse plasmon modes and well-established synthesis and assembly methods.^25–32^ Despite extensive use of Au NRs in photovoltaics and sensing, their ability to enhance the PEC properties of Au NC–based systems remains largely unexplored. To date, only limited studies have addressed plasmon-enhanced luminescence of Au NCs, leaving their plasmon-modified PEC behavior insufficiently understood.^33,34^

Here, a plasmon-enhanced PEC (PE–PEC) platform is introduced that combines Au NCs immobilized on gold electrodes with Au NRs covered with a thin SiO_2_ spacer. This architecture avoids the use of conventional semiconductor materials while enabling strong plasmonic near-field enhancement. The influence of plasmonic coupling on charge carrier generation is investigated using FDTD simulations and wavelength-dependent photocurrent measurements. Furthermore, the analytical performance of the PE–PEC platform has been evaluated using hydrogen peroxide and catechol as model analytes under physiologically relevant buffer conditions, representing reduction and oxidation reactions, respectively. The results demonstrate that plasmonic enhancement significantly improves PEC sensitivity, highlighting the potential of this strategy for advanced biological sensing applications.

## 2 Experimental Section

### 2.1 Reagents and Materials

Silver nitrate (AgNO_3_, ≥99%), sodium borohydride (NaBH_4_, >98%), glutathione (GSH), 1,4-benzenedithiol (BDT, 99%), (3-Aminopropyl)­trimethoxysilane (APTMS, 99%), cetrimonium bromide (CTAB, ≥98%), ascorbic acid (AA, ≥ 99%), sodium salicylate (≥99.5%), sodium silicate (Na_2_SiO_3_, 27%), hexane (anhydrous, 95%), catechol (≥99%), potassium hexacyanoferrate­(III) (K_3_Fe­(CN)_6_, 99%), and gold­(III) chloride hydrate (HAuCl_4_·*x*H_2_O, 99.995%) were procured from Sigma-Aldrich. Ethanol (≥99.8%), hydrochloric acid (HCl, 37%), nitric acid (HNO_3_, 70%), acetone (≥99.9%), hydrogen peroxide (H_2_O_2_, 30% in H_2_O), and toluene (≥99.8%) were obtained from Carl Roth. Phosphate-buffered saline (PBS, 10 mM, pH 7.4) was acquired from Thermo Fisher Scientific. Round cover glasses (12 mm diameter) were purchased from VWR. Gold electrodes (100 nm Au/10 nm Cr on 150 μm glass, *A* = 144 mm^2^) were custom-fabricated by sputtering.

All chemicals were used as received without further purification. Glassware and stir bars were meticulously cleaned with freshly prepared aqua regia (3:1 v/v HCl:HNO_3_) and rinsed thoroughly with Milli-Q water (18.2 MΩ cm) prior to use.

### 2.2 Preparation and Characterization of Nanomaterials

#### 2.2.1 Au Nanoclusters

Au@GSH nanoclusters (Au NCs) were prepared according to previous literature.^35^ In general, 8 mL of 25 mM HAuCl_4_ aqueous solution, 89 mL Milli-Q water, and 3 mL of 100 mM GSH aqueous solution were combined and stirred at 700 rpm (70–75 °C) under reflux for 24 h. The solution transitioned from a dark goldish-orange to colorless within 5 min, eventually yielding a yellow product. To purify the synthesized gold nanoclusters, unreacted l-glutathione and residual chloroauric acid were eliminated via ultrafiltration. The reaction mixture was subjected to centrifugal filtration using 3 kDa molecular weight cutoff filters (UFC9003, Amicon Ultra) at 4000 rpm for 10 min, with the process repeated three times to ensure thorough removal of low-molecular-weight impurities. The resulting purified and concentrated nanocluster solution was subsequently stored at 4 °C for downstream applications. The concentration of Au NCs was measured at 315 μg/mL in terms of elemental gold by inductively coupled plasma mass spectrometry (ICP-MS, Agilent 7000 Series).

Au nanorods (Au NRs) were synthesized via a seed-mediated growth method, following a previously reported method:^36^

#### 2.2.2 Seed Solution Preparation

A CTAB-stabilized Au seed solution was prepared by dissolving 346.6 mg CTAB (0.2 M) in 5 mL of warm Milli-Q water (60–70 °C) in a 20 mL vial. After cooling to 30 °C, the solution was mixed with 5 mL of 0.5 mM HAuCl_4_. Subsequently, 600 μL of fresh-prepared NaBH_4_ (10 mM) was rapidly injected under vigorous stirring (1200 rpm, 2 min), inducing a color change from yellow to brownish-yellow. The seed solution was aged at room temperature for 0.5–2 h before use.

#### 2.2.3 Growth Solution Preparation

A growth solution was prepared by dissolving 0.9 g CTAB and 80 mg sodium salicylate in 25 mL warm Milli-Q water (60–70 °C) in a 100 mL flask. Upon cooling to 30 °C, 600 μL of fresh 4 mM AgNO_3_ aqueous solution was added (while the Ag does not form part of the final Au NRs, the presence of the Ag^+^ ions facilitates the anisotropic growth), and the mixture was left undisturbed at 30 °C for 15 min. Next, 25 mL of 1 mM HAuCl_4_ aqueous solution was introduced, followed by 15 min of slow stirring (400 rpm). Subsequently, 100 μL of 0.064 M ascorbic acid (AA) was added under vigorous stirring for 30 s until the solution became colorless. Finally, 80 μL of the seed solution was injected, stirred briefly (30 s), and the solution was left undisturbed at 30 °C overnight to facilitate the formation of Au NRs. Excess CTAB was removed via two centrifugation cycles (11,000 rpm, 15 min each) in which the Au NRs were precipitated and the supernatant discarded. The Au NR precipitate was redispersed in 12.5 mL Milli-Q water to obtain the final Au NR solution.

#### 2.2.4 Concentration Determination

UV–vis absorption spectroscopy and ICP-MS elemental analysis were used to determine the Au NR concentration. As reported previously,^37^ an absorbance of 1.2 at 400 nm corresponds to an Au^0^ concentration of 0.5 mM. Our measurements (UV–vis absorption and ICP-MS) confirmed this correlation. The cuvette length used is 1 cm.

For UV–vis absorption measurements, a 5-fold diluted Au NR solution was scanned from 190–1100 nm. For ICP-MS sample preparation, 10 μL of the diluted Au NR solution was mixed with 190 μL aqua regia and gently shaken for 2 h to ensure complete dissolution. Subsequently, 1.8 mL of 2% (v/v) HNO_3_ was added, and the sample was aged overnight prior to ICP-MS analysis.

To prepare Au NR@SiO_2_, 10 mL of the purified Au NRs solution was diluted with 30 mL Milli-Q water, followed by the addition of a freshly prepared 0.54 mM APTMS aqueous solution under vigorous stirring for 10 min. This step ensures the functionalization of the Au NRs surface via amine group complexation. Subsequently, a 0.54 wt % sodium silicate aqueous solution (pH 10.9) was introduced, and the mixture was stirred continuously for 24 h to facilitate the formation of an ultrathin SiO_2_ shell around the Au NRs.^38^ The resulting Au NR@SiO_2_ were purified via centrifugation (11,000 rpm, 15 min) and redispersed in 2.5 mL Milli-Q water to yield the final product. For amino-functionalization, APTMS (1 μL) was added to concentrated Au NR@SiO_2_ (1 mL) under continuous shaking for 5 h at room temperature, followed by heating at 50 °C for 1 h. The finally resulting Au NR@SiO_2_ were washed and collected via centrifugation (8000 rpm, 10 min).

The optical properties of the as-prepared nanomaterials were characterized using UV–vis absorption spectroscopy (Agilent 8453 spectrophotometer) and fluorescence spectroscopy (Fluorolog FL3-22). Zeta potential measurements (Malvern Zetasizer Nano ZS, ZEN 3600) were employed to systematically characterize the surface modifications of the nanomaterials. Zeta potential (ζ) measurements of Au NRs, Au NR@SiO_2_, and Au NCs are shown in Figure S4. The morphologies of the nanomaterial samples were analyzed using transmission electron microscopy (TEM, Jeol-JEM 1011 FS, 100 kV) and scanning electron microscopy (SEM, Nova NanoSEM 650, 15 kV).

### 2.3 Fabrication of the PE–PEC Working Electrode

Gold electrodes on the glass chips were prepared by DC sputter deposition in a Cressington 308R coater equipped with two sputter heads (Cr, Au). After mounting the samples upside-down on a rotatable sample holder, the chamber was evacuated overnight, reaching a base pressure of 1 × 10^–6^ mbar. The chamber was then flushed with argon and evacuated again. Finally, by opening a needle valve to the argon line, a process pressure of 0.02 mbar was set. About 10 nm of Cr were deposited as an adhesion promoter with a set current of 40 mA for 2 min; subsequently, without a vacuum break, Au was sputtered at 40 mA for 5 min, leading to a gold film thickness of approximately 100 nm.

These gold chip electrodes (AuE) were cleaned via sequential ultrasonication in acetone, absolute ethanol, and Milli-Q water. BDT (50 mM in ethanol) was used to form a (mono-) layer by immersion for 4 h. BDT-modified electrodes (S-AuE) were rinsed with ethanol and dried under N_2_. For the characterization of such layers, we refer to a previous publication.^39^

Au@GSH NCs were deposited onto the S-AuE chips for 5 min, followed by spin-coating to remove excess solvent, yielding the normal PEC electrodes.

A (mono-) layer of Au NR@SiO_2_ was formed at a hexane/water interface by adding hexane (230 μL) and ethanol (1.8 mL) to the nanoparticle solution (1.5 mL). After hexane evaporation, the formed film was transferred onto AuNC/S-AuE via a floating transfer process,^40^ ultimately fabricating the PE–PEC sensing platform.

All electrodes were fabricated following identical procedures with controlled deposition and assembly parameters (e.g., precursor concentration, incubation time, and transfer conditions) to ensure reproducibility and comparability between different samples.

### 2.4 PEC Measurement

Photoelectrochemical measurements were performed using a custom-built PEC system housed in a Faraday cage (Figure S1). A 300 W Xe-arc lamp (Ushio, Inc., Japan) provided broadband illumination (250–1100 nm), modulated at 71 Hz with an optical chopper (Scitec Instruments, USA) and controlled by an LPS-220 power supply (Photon Technology International, USA). Photocurrent signals were amplified by an EG&G 5210 lock-in amplifier and recorded via LabVIEW. In order to ensure consistency in the data, a minimum of three independent electrodes has always been measured.

Measurements were carried out in a three-electrode configuration using the nanostructured chip as the working electrode (WE), an Ag/AgCl (3 M NaCl) reference electrode (RE), and a Pt wire counter electrode (CE). Illumination was manually controlled with a mechanical shutter.

The electrochemical cell featured a 6 mm diameter optical window (*A*_chip_ ≈ 0.28 cm^2^), with the WE sealed using a Teflon fitting and O-ring. Electrical contact was established via conductive tape (Figure S2).

Spectral response was evaluated using bandpass filters: 405 ± 10 nm (BrightLine), 445 ± 50 nm (Carl Zeiss), 525 ± 50 nm (Carl Zeiss), 530–585 nm (Carl Zeiss), 580 ± 25 nm (CHROMA), 605 ± 50 nm (CHROMA), 640 ± 25 nm (CHROMA), and 725 ± 50 nm (Carl Zeiss) and radiant flux calibrated using a FieldMax-II optical power meter.

Before analyte detection (H_2_O_2_ and catechol), the electrochemical cell and all electrodes (WE, CE, and RE) were rigorously rinsed with Milli-Q water and dried under N_2_ flow. Both analyte solutions were prepared in 10 mM phosphate-buffered saline (PBS, pH 7.4) at specified concentrations. All electrochemical measurements were performed under deaerated conditions by purging the electrolyte with nitrogen for 20 min prior to analysis.

Photocurrent measurements were acquired in 10 mM phosphate-buffered saline (PBS, pH 7.4) at an applied potential of *U* = −0.2 V vs Ag/AgCl using light excitation at wavelength λ. The incident light intensity was normalized to 114 mW·cm^–2^ for all wavelengths using calibrated optical filters and verified with a reference photometer.

Electrochemical impedance spectroscopy (EIS) measurements were conducted over a frequency range of 0.1 Hz to 100,000 Hz at a fixed potential of 0 V versus the OCP, using a sinusoidal excitation signal with an amplitude of 10 mV. The experiments were performed in 10 mM phosphate-buffered saline (PBS) containing a 5 mM [Fe­(CN)_6_]^3–^/[Fe­(CN)_6_]^4–^ redox couple to investigate the interfacial properties of the electrode surface.

The FDTD simulations were conducted using the open-source MEEP software.^41^ The simulations encompassed both, a single Au NR@SiO_2_ and an assembly of four such nanorods. Each Au nanorod measured 40 nm in length and 20 nm in width, with a uniform SiO_2_ shell thickness of 2 nm. Excitation wavelengths of 532 and 672 nm were employed to simulate responses in the transverse and longitudinal directions, respectively. For UV–vis absorption spectrum simulation, a continuous wavelength range from 400 to 800 nm was utilized.

## 3 Results and Discussion

### 3.1 Characterization of Nanomaterials and Electrode Preparation

Scheme 1 illustrates the fabrication of the PE–PEC sensor. First, a thiol compound (BDT) was immobilized on the gold electrode (AuE) surface via chemisorption, yielding a dithiol-functionalized electrode (S-AuE; Scheme 1c). The Au NCs were then deposited onto the S-AuE by spin coating to form a conventional Au NC-based PEC electrode. Subsequently, a monolayer of Au NRs coated with a thin SiO_2_ spacer layer (Au NR@SiO_2_) was transferred onto the Au NC-modified PEC electrode via a floating transfer process, resulting in the PE–PEC sensing platform (Scheme 1d). The structural and optical properties of Au NCs, Au NRs, and Au NR@SiO_2_ have been characterized by absorption spectroscopy, fluorescence measurements, and transmission electron microscopy (TEM). As shown in Figure S3, the Au NCs exhibit a core diameter of *d*_c_(Au NCs) = 1.9 ± 0.1 nm, consistent with the previous report.^42^ TEM images (Figure 1a,b) reveal the rather uniform Au NRs with an average diameter of *d*_c_(Au NRs) = 20.2 ± 1.5 nm and a length of *l*_c_(Au NRs) = 39.4 ± 2.4 nm, while Figure 1b confirms the presence of a thin SiO_2_ shell with a thickness of Δ_s_ = 1.9 ± 0.3 nm (Figure 1b). The UV–vis absorption spectrum of the Au NRs displays characteristic transverse (LSPR_T_) and longitudinal (LSPR_L_) peaks at 520 and 605 nm, respectively (Figure 1c).

**Figure 1:**
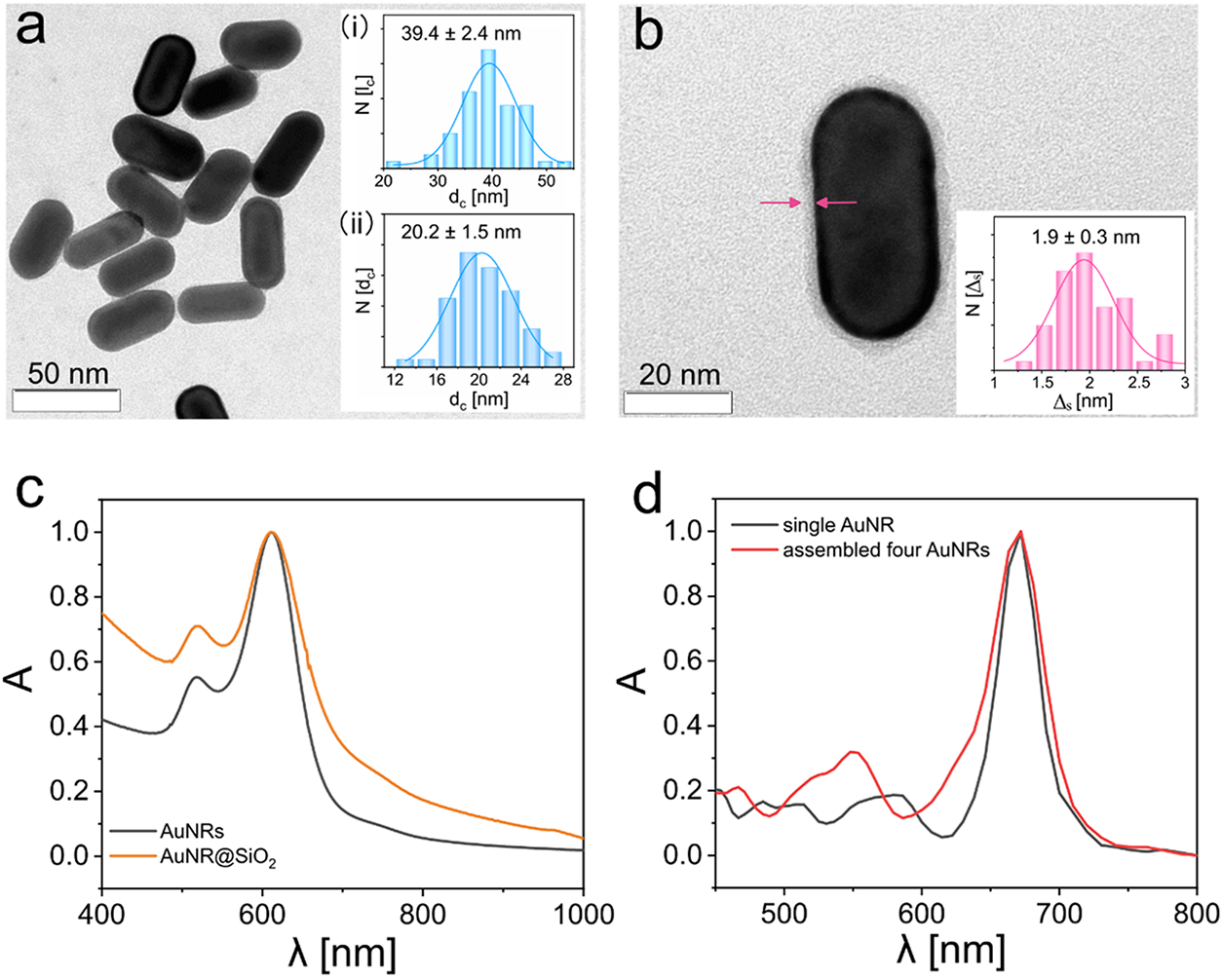
Characterization of Au NRs and Au NR@SiO_2_. (a) Typical TEM image and (i) transverse and (ii) longitudinal size distributions of Au NRs. N­(*d*_*c*_) and N­(*l*_*c*_) refer to the number of counts of NPs with a core diameter *d*_*c*_ and a length *l*_*c*_, respectively. (b) TEM image of Au NR@SiO_2_ and corresponding thickness (Δ_*s*_) distribution of silica with 1.9 ± 0.3 nm. (c) Normalized UV–vis absorption *A*(λ) of Au NRs and Au NR@SiO_2_. (d) Normalized simulated UV–vis absorption *A*(λ) of a single Au NR@SiO_2_ and assembled four Au NR@SiO_2_ by FDTD simulation.

**Scheme 1:**
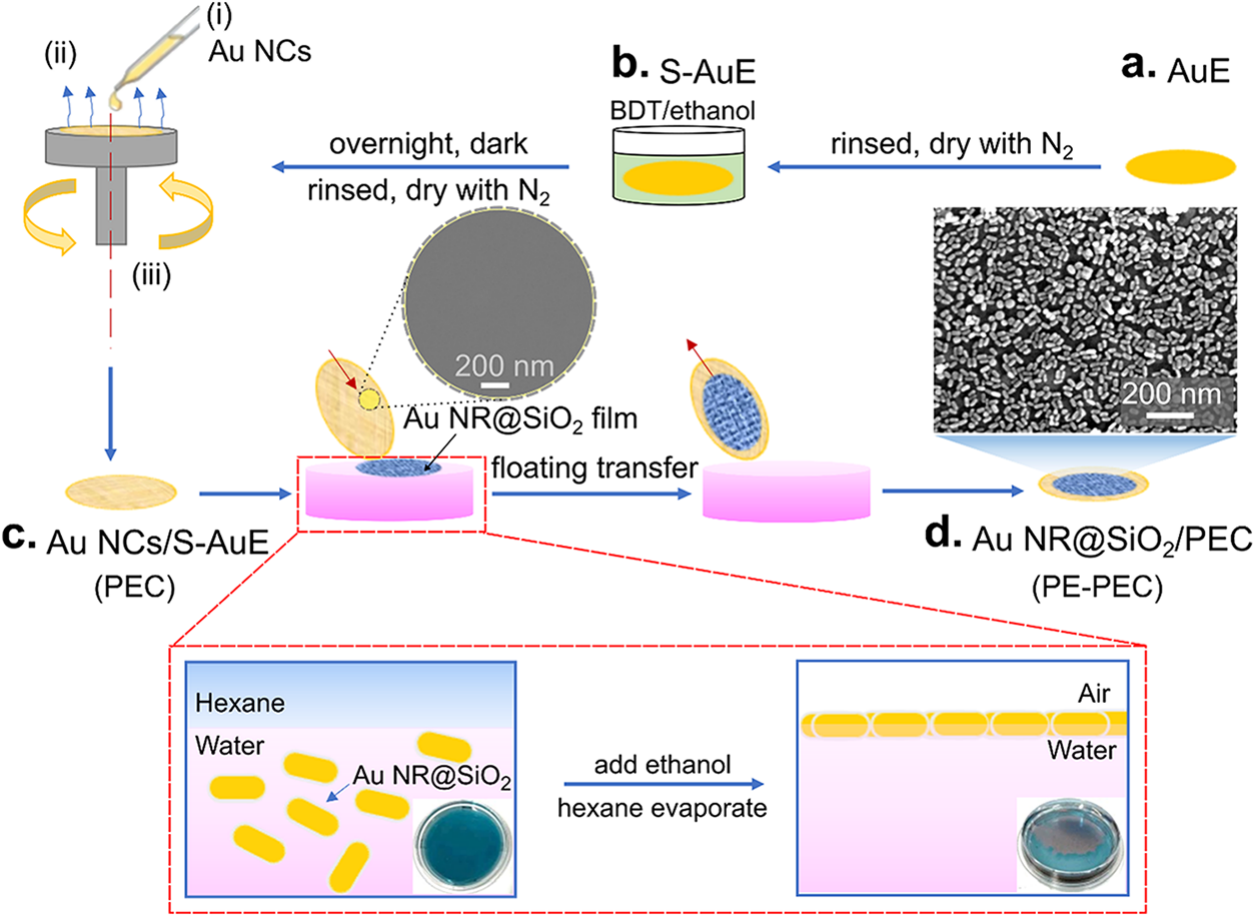
Schematics of the Preparation of the PE–PEC Platform via a Layer-by-Layer Process^a^

a (a) start point is a clean gold electrode (AuE), (b) this electrode is modified by benzene dithiol (S-AuE), (c) gold nanoclusters are fixed onto this thiol layer (Au NC/S-AuE = PEC) as shown with a SEM image of the PEC electrode (A spinning coating process was used to immobilize the Au@GSH NCs to obtain normal PEC, which consists of (i) deposition, (ii) rotating, and (iii) evaporating steps), and (d) gold nanorods with a defined SiO_*2*_ layer are immobilized on top (Au NR@SiO_*2*_/PEC = PE–PEC electrode). SEM images of PE–PEC suggest the successful immobilization of Au NR@SiO_*2*_. The Au NR@SiO_*2*_ film was formed by self-assembling Au NR@SiO_*2*_ at the water/hexane interface by adding ethanol and evaporating the hexane (bottom row) and finally transferring the layer onto the PEC electrode. The inset represented photographs of the Au NR@SiO_*2*_ solution (left) and the Au NR@SiO_*2*_ monolayer formed at an air/water interface (right).

The successful immobilization of Au NCs and Au NRs@SiO_2_ was verified by UV–vis absorption spectroscopy using glass substrates in place of metal electrodes (Figure S5a). The immobilized Au NCs retained spectral features comparable to those observed in an aqueous solution, confirming their successful deposition without significant alteration of their optical properties (Figure S5b). The solid-state Au NRs@SiO_2_ exhibit a broader absorption band compared to the aqueous dispersion, which can be attributed to partial aggregation on the glass surface (Figure S5c). The electrode architecture was further examined by scanning electron microscopy (SEM) (Scheme 1d, inset), which reveals a relatively uniform distribution of Au NRs@SiO_2_ on the PE–PEC platform. The small interparticle distance suggests strong plasmonic coupling between neighboring nanorods, leading to a pronounced enhancement of the electromagnetic near-field compared to isolated Au NR@SiO_2_. Such local field enhancement is known to depend strongly on the nanostructure’s geometry and the surrounding environment.^43–45^ To estimate the loading of Au NRs on the electrode surface, a semiquantitative analysis was conducted based on SEM images. By counting the number of nanorods in several representative regions, the particle density was calculated to be on the order of ∼800 rods μm^–2^. Based on the average dimensions of the nanorods (length ∼40 nm and width ∼20 nm), the projected area was approximated assuming an elliptical geometry, resulting in an estimated surface coverage of ∼60–70%. It should be noted that this value is derived from localized observations and simplified assumptions, and thus represents a semiquantitative estimation illustrating the order of magnitude. Direct determination via elemental analysis of the Au NRs by their Au content could not be carried out, as also the underlying electrode structure contains Au.

FDTD simulations were performed for isolated and assembled Au NRs (Figure 2) to unveil the extent of local field enhancement.^22^ For single nanorods, the electromagnetic (EM) field intensity at the surface is enhanced by approximately 15-fold under longitudinal excitation (Figure 2a) and 6-fold under transverse excitation (Figure 2c). In contrast, assemblies of four closely spaced Au NRs with an interparticle gap of 4 nm exhibit dramatically increased near-filed enhancement, exceeding 150-fold and 30-fold for longitudinal and transverse modes, respectively (Figure 2b,d).

**Figure 2:**
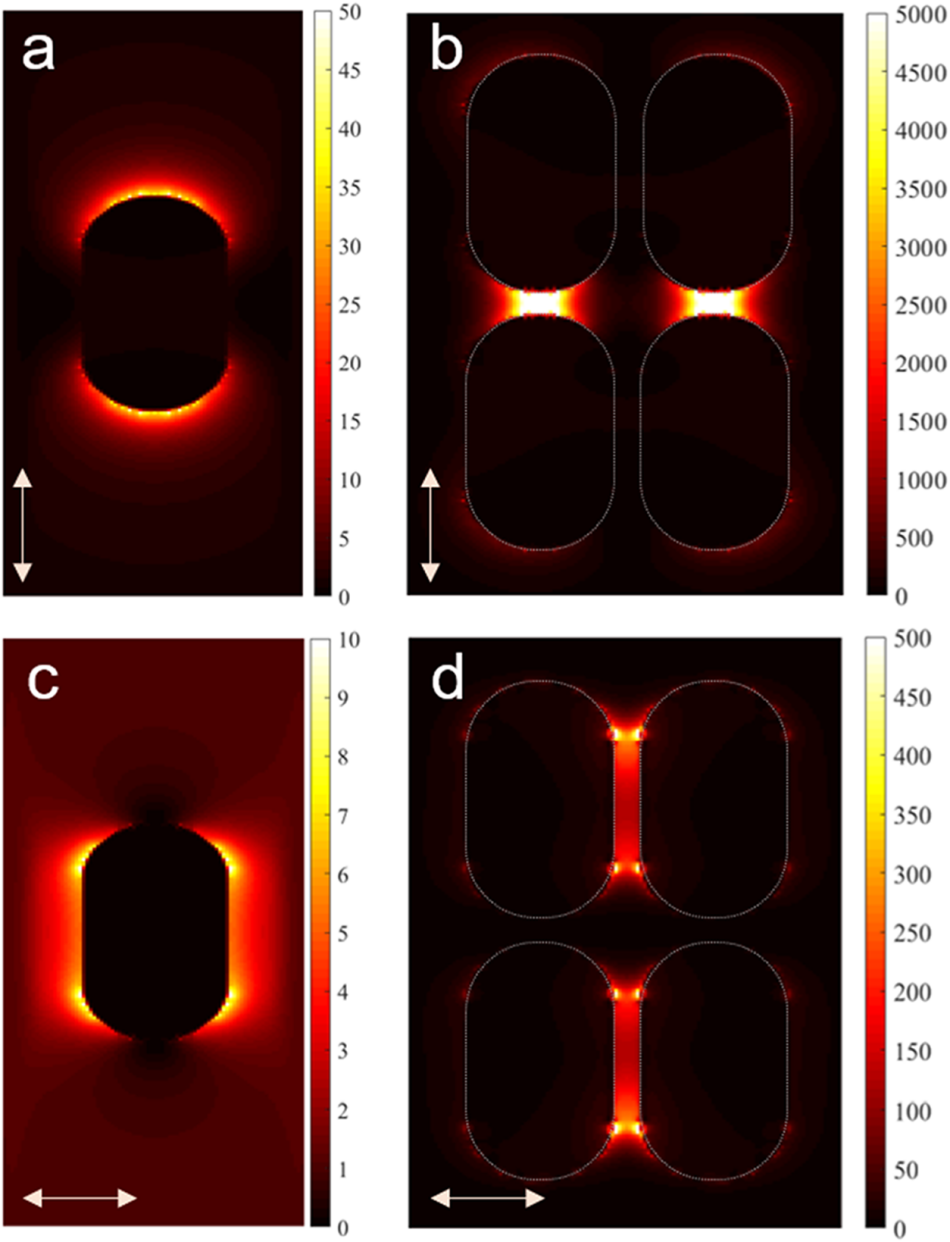
FDTD simulation results of the normalized EM field intensity distribution (|E^2^|/|E0|) from the XZ plane for the isolated Au NR@SiO_2_ (a, c) and four assembled Au NRs@SiO_2_ (b, d) in longitudinal (a, b) and transversal direction (c, d), respectively. The size of Au NR was *l*_c_ = 40 and *d*_c_ = 20 nm in length and width, respectively, and the SiO_2_ coating was set at Δ_s_ = 2 nm. The arrows indicate the excitation polarization. The shadow line indicates the position of the single Au NR@SiO_2_. The colors from black to yellow represent the EM field intensity from weak to strong. |E2| and |E0| stand for the scattered EM-field intensity and the incident EM-field intensity, respectively.

In addition to the stronger field confinement, the simulated spectra of the assembled nanorods show broader resonance features compared to isolated Au NRs (Figure 1d). This broadening arises from plasmonic coupling between adjacent nanorods and indicates an extended wavelength-dependent electromagnetic response. It is noted that the simulated LSPR positions are red-shifted relative to experimental UV–vis spectra (Figure 1c). This discrepancy is attributed to the idealized geometry, periodic arrangement, and homogeneous dielectric environment assumed in the simulations compared to the experimentally distributed nanorod ensemble. Such differences between simulated and experimental plasmonic responses are widely reported in the literature.^46,47^ Accordingly, the simulations are primarily used to qualitatively capture plasmonic trends rather than exact resonance positions.

The plasmonic near-field is known to decay rapidly with distance from the metal surface; therefore, maintaining a nanometer-scale spacer is essential for effective coupling. In this work, a ∼2 nm SiO_2_ layer provides a reasonable balance between strong electromagnetic enhancement and suppression of direct interfacial charge transfer between Au NRs and Au NCs. Additional FDTD simulations performed with SiO_2_ spacer thicknesses ranging from 2 to 8 nm further confirm that the local EM field intensity decreases significantly as the spacer thickness increases (Figure S6), indicating progressively weakened plasmonic coupling at larger separation distances. Although a systematic investigation over a broader thickness range could help to identify an optimal spacer thickness, such optimization is beyond the scope of the present proof-of-concept study.

### 3.2 Photoelectrochemical Properties of the PEC- and the PE-PEC Systems

In the next step, the photoelectrochemical properties of the Au NCs/Au NRs hybrid system were characterized. Wavelength-dependent photocurrent measurements (Figure 3a) reveal that Au NR@SiO_2_-modified electrodes exhibit consistently higher photocurrent responses compared to the Au NCs-only electrodes across the entire spectral range. Two distinct photocurrent maxima are observed at ∼525 and ∼615 nm, which coincide with the LSPR_T_ and LSPR_L_ absorption bands of Au NRs@SiO_2_, respectively. This clear spectral correspondence indicates that the photocurrent enhancement is strongly governed by plasmonic excitation of the Au NRs. The underlying mechanism can be rationalized as follows: Au NCs exhibits optical absorption only at wavelengths below 500 nm (Figure S3); therefore, illumination at longer wavelengths does not directly excite the Au NCs and does not result in intrinsic charge-carrier generation. Consequently, the photocurrents observed under illumination with wavelengths above 500 nm can be attributed primarily to the presence of Au NRs in the PE–PEC architecture. Interestingly, the photocurrent enhancement at 525 nm is more pronounced than at 615 nm, despite both wavelengths corresponding to LSPR modes. This indicates that strong local-field enhancement alone does not necessarily translate into higher photocurrent generation. Such behavior is consistent with previous studies on Au NRs, which reported a lower quantum efficiency for the LSPR_L_ mode compared to the LSPR_T_ mode.^48^

**Figure 3:**
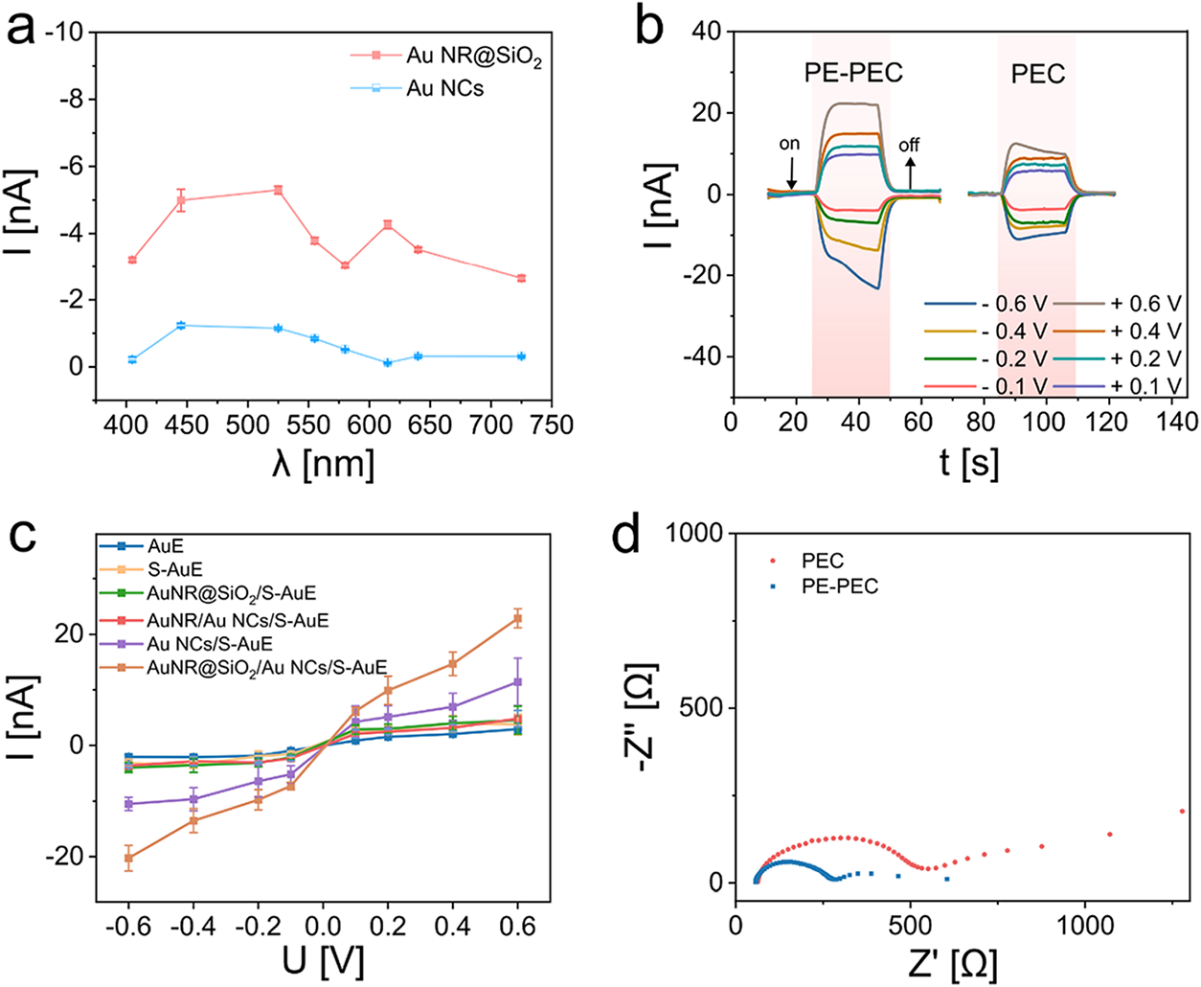
(a) Wavelength-dependent rectified photocurrent I of the Au NRs@SiO_2_ and Au NC coated sensor, i.e., Au NR@SiO_2_/Au NC/S-AuE and Au NC/S-AuE. (b) Traces of the current responses I (t) as recorded with the PEC and PE–PEC electrodes in the absence and presence of white light illumination at varying electrode potential U vs Ag/AgCl. (c) Photocurrent I in dependence on the applied voltage U for AuE, S-AuE, Au NC/S-AuE (PEC), and Au NR@SiO_2_/Au NC/S-AuE (PE–PEC) compared with Au NR@SiO_2_/S-AuE (in the absence of Au NCs) and Au NR/Au NC/S-AuE (in the absence of a spacer SiO_2_-layer on the Au NRs) electrodes. (d) EIS of PEC (Au NC/S-AuE), and PE–PEC (Au NR@SiO_2_/Au NC/S-AuE) electrodes under white light illumination in 5 mM hexacyanoferrate solution, showing the real and the imaginary part of the impedance (*Z′* and *Z″*). 10 mM PBS buffer (pH 7.4) was used in these experiments. Error bars represent the standard deviation of three measurements.

To further evaluate the interfacial photoelectrochemical behavior, potential-dependent photocurrent (I) measurements were performed (Figure 3b,c). The PE–PEC electrode consistently shows higher photocurrent responses compared to all control configurations, including bare AuE, S-AuE, and Au NR@SiO_2_/S-AuE (Figure 3c). At *U* = −0.6 V vs Ag/AgCl, the PE–PEC (Au NR@SiO_2_/Au NC/S-AuE) electrode reaches a photocurrent of *I* = −20.25 nA, which is approximately 1.93 times higher than that of the normal PEC electrode (Au NC/S-AuE: *I* = −10.5 nA) and 5 times higher than that of Au NR@SiO_2_/S-AuE (*I* = −4.0 nA).

This analysis shows that the photocurrent of the PE–PEC system (|*I*| = 20.25 nA) exceeds the sum of the individual components. Assuming comparable surface coverage of Au NRs@SiO_2_ in the relevant electrode configurations, this enhancement can be attributed to an increased charge-carrier generation within the Au NCs induced by their interaction with the plasmonic nanorods. Furthermore, removal of the SiO_2_ spacer layer leads to a pronounced decrease in photocurrent; for example, at *U* = −0.6 V vs Ag/AgCl, the photocurrent drops from *I* = −20.25 nA for Au NR@SiO2/Au NC/S-AuE to *I* = −3.6 nA for Au NR/Au NC/S-AuE, highlighting the critical role of a nanoscaled separation in regulating interfacial interactions. This observation suggests that direct charge transfer between AuNCs and AuNRs is unfavorable for efficient photocurrent generation. This might lead to the argument that energy transfer is mainly behind the enhancement effect and not electron transfer, since the latter would be hindered (though due to porosity not completely suppressed) by the SiO_2_ shell. On the other hand, in this argumentation it is assumed that the amount of Au NRs in the Au NR@SiO_2_/Au NC/S-AuE and Au NR/Au NC/S-AuE geometry is the same. This is rather reasonable since a floating technique has been used for the Au NR deposition diminishing the effect of the underlying surface. Within the framework of this study it was not possible to experimentally determine the exact amount of Au NRs on the sensor surface for the different geometries. This gives some limitations in the precise mechanism, but the wavelength-dependent measurements clearly demonstrate the enhancement in photocurrent by the nanorods introduced.

Additionally, EIS was utilized to investigate the interfacial charge-transfer properties of the PEC and PE–PEC electrodes under both illuminated (Figure 3d) and nonilluminated conditions (Figure S7). In both cases, the Nyquist plots exhibit a typical semicircular shape in the high-frequency region, which can be attributed to the parallel circuit of the interfacial charge-transfer resistance and the double-layer capacitance. A smaller radius of the semicircle refers to a lower charge-transfer resistance and thus, a more efficient charge transport across the electrode–electrolyte interface (redox reaction of the chosen redox couple). Under illumination, the PE–PEC electrode displays a noticeably smaller semicircle compared to the conventional PEC electrode, indicating a significantly reduced charge-transfer resistance. This observation is consistent with the enhanced photocurrent response observed for the PE–PEC system and confirms the beneficial effect of incorporating Au NRs@SiO_2_ into the electrode architecture. In dark conditions, both electrodes show significantly higher resistance (Figure S7), confirming the light-dependent modulation of interfacial processes. Based on these combined results, the enhanced photocurrent in the PE–PEC system can be attributed to plasmon-driven near-field interaction between Au NRs and Au NCs, which enhances charge-carrier generation within the nanocluster layer. Contributions from additional interfacial effects cannot be fully excluded due to the complexity of the system, but mechanisms such as hot electron injections or plasmon-induced resonance energy transfer (PIRET) can rather be excluded as dominant mechanism because of the insulating nature of the SiO_2_ layer on the one hand and the limited spectral overlap between Au NC and Au NR on the other hand.

### 3.3 Response Study of Selected Model Analytes

Finally, the sensing performance toward the model reaction of H_2_O_2_ reduction was evaluated under white-light illumination (157 mW cm^–2^). Previous studies indicated that Au NCs-based PEC systems can detect H_2_O_2_ conversion under negative polarization.^10^ In the present study various negative potentials (from −0.1 to −0.6 V vs Ag/AgCl) were applied for H_2_O_2_ detection. In order to compare the results of the PE–PEC geometry versus a PEC electrode (Au NCs alone), both electrodes have been studied in the absence and presence of H_2_O_2_. Original current–time profiles are given in Figure S8 and photocurrents are plotted in Figure 4. As demonstrated in Figure 4a,b, a significantly lower cathodic current response was observed for the PEC electrode. In contrast, the defined increase in photocurrent of the PE–PEC system upon H_2_O_2_ addition indicates the capability of the combined electrode for H_2_O_2_ detection.

**Figure 4:**
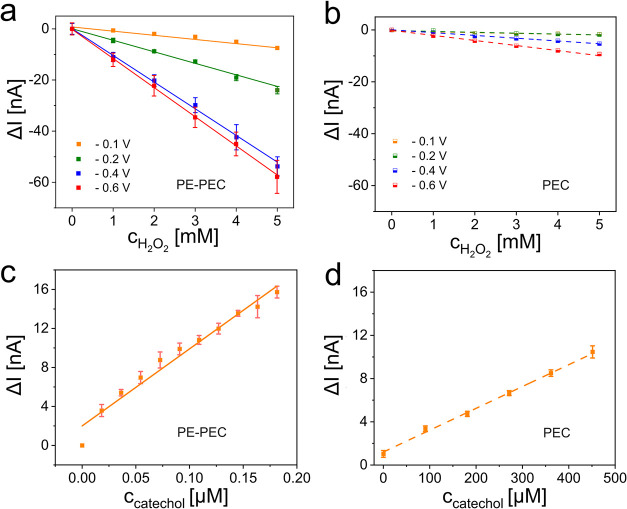
Response properties of the electrodes: (a–b) Photoelectrochemical detection of H_2_O_2_: Photocurrent response (Δ*I*) as a function of H_2_O_2_ concentration (*c*_H_2_O_2__) and applied potential (U, versus Ag/AgCl) for (a) PE–PEC and (b) PEC electrodes. These measurements were collected in 0.01 M PBS buffer (pH 7.4) under white light illumination. (c–d) Photoelectrochemical detection of **catechol**: Photocurrent response (Δ*I*) as a function of catechol concentration (*c*_catechol_) (under applied voltage of *U* = 0 V) for (c) PE–PEC and (d) PEC electrodes. Error bars represent the standard deviation of repeated measurements (*N* = 3).

The observed experimental effects indicate that energy transfer plays a dominant role in enhanced photocurrent generation in the PE–PEC system. This is suggested by the absence of large photocurrents when the Au NRs are used alone on the gold electrode. In addition, the SiO_2_ layer on the Au NRs is not beneficial for electron transfer reactions and also not for reactions of hydrogen peroxide, due to its insulating nature. Consequently, the enhanced charge carrier generation within the NCs seems responsible for the enhanced hydrogen peroxide conversion at the PE–PEC electrode, which, however, remains an evidence rather than a proof.

To characterize the analytical potential of the PE–PEC system, the photocurrent responses of PE–PEC and conventional PEC electrode have been investigated at varying H_2_O_2_ concentrations. As depicted in Figure 4a,b, the sensitivity of both the PE–PEC and the PEC electrodes increases with applied negative potential. The PE–PEC architecture, however, exhibits a higher sensitivity compared to the PEC system alone, i.e., the same H_2_O_2_ concentration leads to a larger change in photocurrent (Δ*I*), defined as Δ*I* = *I*–*I*_0_, where *I* and *I*_0_ represent the steady-state photocurrent in the presence and absence of H_2_O_2_, respectively, recorded under identical illumination and at a given potential. The PE–PEC electrode exhibits a limit of detection (LOD) of 6.6 μM (at −0.6 V), which is lower than the normal PEC with an LOD of 43 μM at the same concentration (Figure 4a,b). This indicates that the PE–PEC platform is superior to traditional PEC sensors due to its ability to detect H_2_O_2_ with a higher response (Table S1). Interestingly, the sensitivity of the PE–PEC detection still increases with the applied potential, which can be mainly attributed to a more efficient separation of electron–hole pairs.

It should be noted that the absolute surface coverage of Au NCs and Au NRs is difficult to quantify due to the all gold nature of the nanostructures on the electrodes. However, this limitation does not impede the validity of the comparative performance analysis, since all electrodes were fabricated using identical deposition conditions, including precursor concentration, incubation time, and processing protocol. Therefore, the observed differences in photocurrent response can be attributed to the presence of Au NRs rather than variations in loading amount. This statement is also supported by reproducibility measurements as given in Figure S11, which demonstrate RSDs below 5% for repeatedly produced electrode systems.

The capability of the PE–PEC electrode to detect additional analyte substances under light irradiation has been further evaluated using catechol as a model compound. In contrast to hydrogen peroxide, which undergoes reduction under negative bias potential, catechol is oxidized at the electrode surface, resulting in anodic photocurrents. Both the PE–PEC and the conventional PEC systems were investigated for comparison. Representative current–time responses recorded upon various concentrations of catechol are shown in Figure S9a,b, while the corresponding photocurrent values are summarized in Figure 4c,d. Both electrode configurations exhibit a measurable response toward catechol; however, the PE–PEC displays substantially larger photocurrent changes with increasing catechol concentration. Consequently, significantly lower analyte concentrations are required to generate photocurrent responses in the nanoampere range for the PE–PEC system compared to the PEC electrode. This behavior demonstrates that the higher photocurrent generation at the PE–PEC electrode is accompanied by a better detection capability of a donor molecule in solution. Thus, low concentrations of catechol can be detected using the PE–PEC platform without the need for biochemical amplification strategies.^49,50^ A linear relationship between the photocurrent signal I and catechol concentration is obtained in the range of 0.018–0.18 μM for the PE–PEC sensor, and 0.27–450 μM for the PEC sensor, with a correlation coefficient (R^2^) of 0.995 and 0.97, respectively. Although the intention here was not to develop a complete sensor, the analytical characteristics have been compared with some examples from the literature as given in Table S2. The sensitivity of the PE–PEC system is enhanced by orders of magnitude compared to the PEC electrode. Control experiments using Au NR@SiO_2_/S-AuE and bare AuE electrodes show only minor or negligible photocurrent responses over the tested concentration ranges, confirming that the pronounced signal amplification originates from the combined Au NC-Au NR@SiO_2_ photoelectrode architecture (Figure S9c,d). While the detailed origin of this strong signal enhancement for catechol cannot yet fully be resolved, the presented results clearly demonstrate that the PE–PEC geometry allows for a sensitive and rapid detection of catechol under controlled buffer conditions. A plausible sensing mechanism is proposed based on the photoelectrochemical oxidation reaction of catechol, which leads to electron injection into the Au NCs. This process is expected to reduce charge-carrier recombination within the Au NCs, thereby enabling higher steady-state photocurrents (Scheme 2), in agreement with previous reports on phenolic compound detection.^51^

**Scheme 2:**
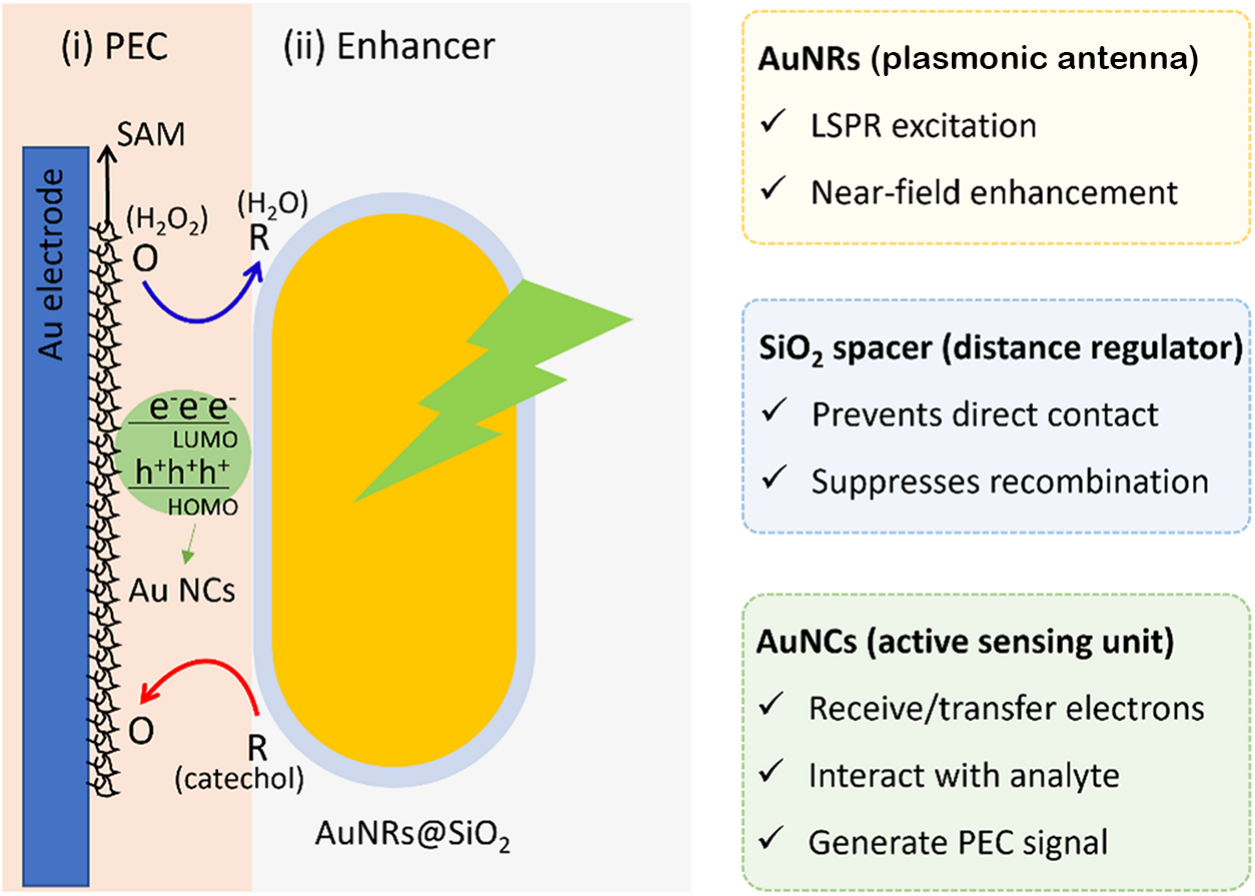
Schematic Illustration of the PE–PEC Hybrid System, the Corresponding Plasmon-Enhanced Charge-Transfer Process and the Main Function of the Components

The increased charge-carrier generation in the PE–PEC system could be used to enhance the detection of the phenolic substance with much higher conversion rates compared to hydrogen peroxide. Additional effects contributing to the enhanced response cannot be ruled out. Catechol is nonpolar; therefore, the different charges of the used nanoparticles (positively charged Au NRs@SiO_2_ and negatively charged Au NCs) are unlikely to play a role. Catechol, however, might be preconcentrated in the pores of the SiO_2_ shells, leading to a locally increased analyte concentration and thus, enhanced photocurrent responses.^52,53^

Finally, the operational stability of the PE–PEC electrode has been evaluated under continuous illumination over 500 s for both H_2_O_2_ reduction and catechol oxidation. Stable photocurrent responses without noticeable signal decay in either case have been found (Figure S10), indicating robustness of the electrode under the applied sensing conditions.

The analytical performance of the PE–PEC sensor has so far been evaluated exclusively in buffer solution. While the results demonstrate high sensitivity for both photoelectrochemical reduction (H_2_O_2_) and oxidation (catechol) reactions, further studies addressing selectivity, operational stability, and performance in complex real-world matrices—such as biological fluids or environmental samples—are required to fully assess the practical applicability of the sensing platform. Here, we demonstrate the general feasibility of the concept of a semiconductor-free sensing electrode with proper sensitivity by plasmonic enhancement. The observed increase in signal generation across fundamentally different reaction types highlights the broad potential of the PE–PEC architecture as a versatile photoelectrochemical sensing strategy.

## 4 Conclusion

A plasmon-enhanced photoelectrochemical (PE–PEC) platform based on gold nanoclusters (Au NCs) and plasmonic gold nanorods (Au NRs) coated with a thin SiO_2_ spacer layer has been successfully developed. This all-gold architecture eliminates the need for semiconductor photoactive materials while achieving significantly enhanced photocurrent generation under visible light illumination. Combined theoretical simulations and wavelength-dependent experiments suggest that near-field induced energy transfer between Au NRs and Au NCs play a dominant role in the observed enhancement, although direct spectroscopic evidence is not provided in this study. Potential-dependent measurements verify the feasibility for enhanced cathodic and anodic photoresponses.

The analytical performance of the PE–PEC system was demonstrated with hydrogen peroxide and catechol as model analytes under physiologically relevant conditions. In both cases, the PE–PEC platform exhibits substantially higher sensitivity compared to conventional Au NC-based PEC electrodes, enabling detection of lower concentrations and waiving the need of biochemical amplification schemes. As a proof-of-concept study, this work focuses on establishing the plasmonic enhancement mechanism and fundamental sensing capabilities of the PE–PEC platform. A comprehensive sensor development including a selectivity analysis in complex sample matrices is beyond the scope of the present study.

Overall, these findings establish plasmonic Au NRs as effective enhancers for Au NC-based PEC systems and provide a general strategy for designing sensitive, semiconductor-free PE–PEC sensors for redox-active species.

## Supplementary Material



## Data Availability

The data supporting this article have been included as part of the Supporting Information.

## Notes

PEC measurement system configuration; material characterization analysis of Au NCs and Au NRs, including zeta potential and optical characterization of functionalized electrodes; EIS measurements; FDTD simulations, PEC performance evaluation; stability and reproducibility analysis of PE–PEC electrodes; and supporting references (PDF)
