# Supplementary material for: Gold Plasmophores for Plasmon-Enhanced Photoelectrochemical Sensing Based on Metal Nanostructures
Source: J Phys Chem A. 2026 Jul 24;130(31):6001–11. doi: 10.1021/acs.jpca.6c01457 (PMC13455101; doi:10.1021/acs.jpca.6c01457)
Supplement: Supplementary file 1 [file jp6c01457_si_001.pdf]

# Gold Plasmophores for Plasmon Enhanced Photoelectrochemical Sensing based on Metal Nanostructures

Lizhen Chen,[1] Robert R. Zierold,[1] Miao Feng,[1] Stefan Werner,[2] Gero Göbel[3], Robert H. Blick,[1] Wolfgang J. Parak,[1,*] Fred Lisdat,[3,*] Indranath Chakraborty[1,4,*]

[1] Fachbereich Physik, Center for Hybrid Nanostructures (CHyN), Universität Hamburg, 22761 Hamburg, Germany

[2] Fachbereich Chemie, Universität Hamburg, Hamburg 20146, Germany

[3] Biosystems Technology, Institute for Applied Life Sciences, Technical University, D-15745 Wildau, Germany

[4] School of Nano Science and Technology, Indian Institute of Technology Kharagpur, Kharagpur-721302, India

*corresponding author: indranath@iitkgp.ac.in, wolfgang.parak@uni-hamburg.de, flisdat@th-wildau.de

# Table of Contents

S1 PEC measurement system configuration.......... S3
S2 Characterization analysis.......... S4
S2.1 Characterization of Au NCs.......... S4
S2.2 Zeta Potential (ζ) analysis of Au NCs and Au NRs.......... S5
S2.3 Optical characterization of nanomaterial-functionalized electrodes.......... S6
S3 FDTD simulation.......... S7
S4 PEC measurement.......... S8
S4.1 EIS measurements.......... S8
S4.2 Characterization of PE-PEC performance.......... S9
S4.3 Stability and reproducibility of PE-PEC electrodes.......... S10
S5 References.......... S12

# S1 PEC measurement system configuration

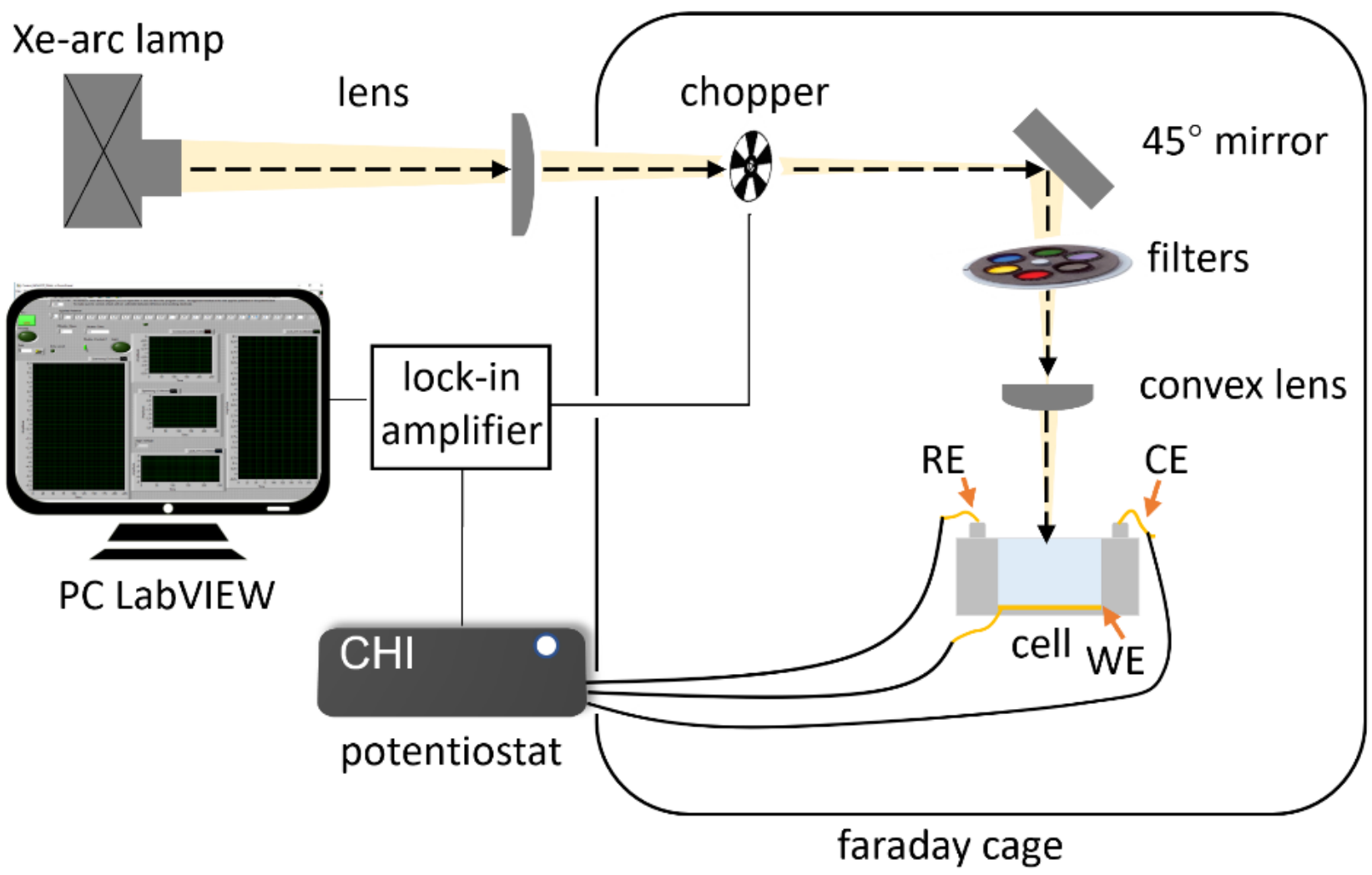


***Fig. S1.*** *Schematic diagram of the homebuilt PEC measurement system.*

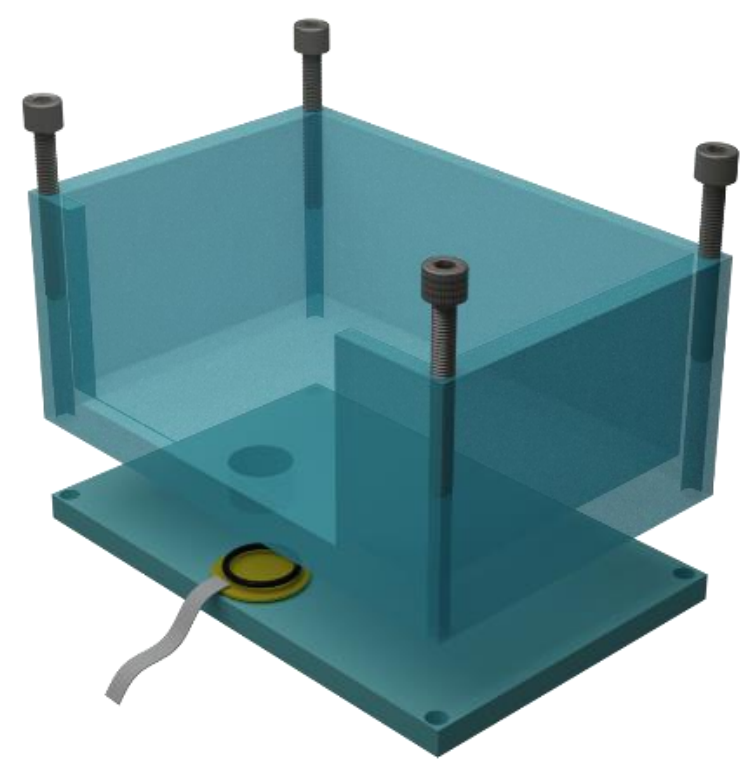

***Fig. S2.*** *Sketch of the electrochemical cell used for the PEC measurements.*

# S2 Characterization analysis

## S2.1 Characterization of Au NCs

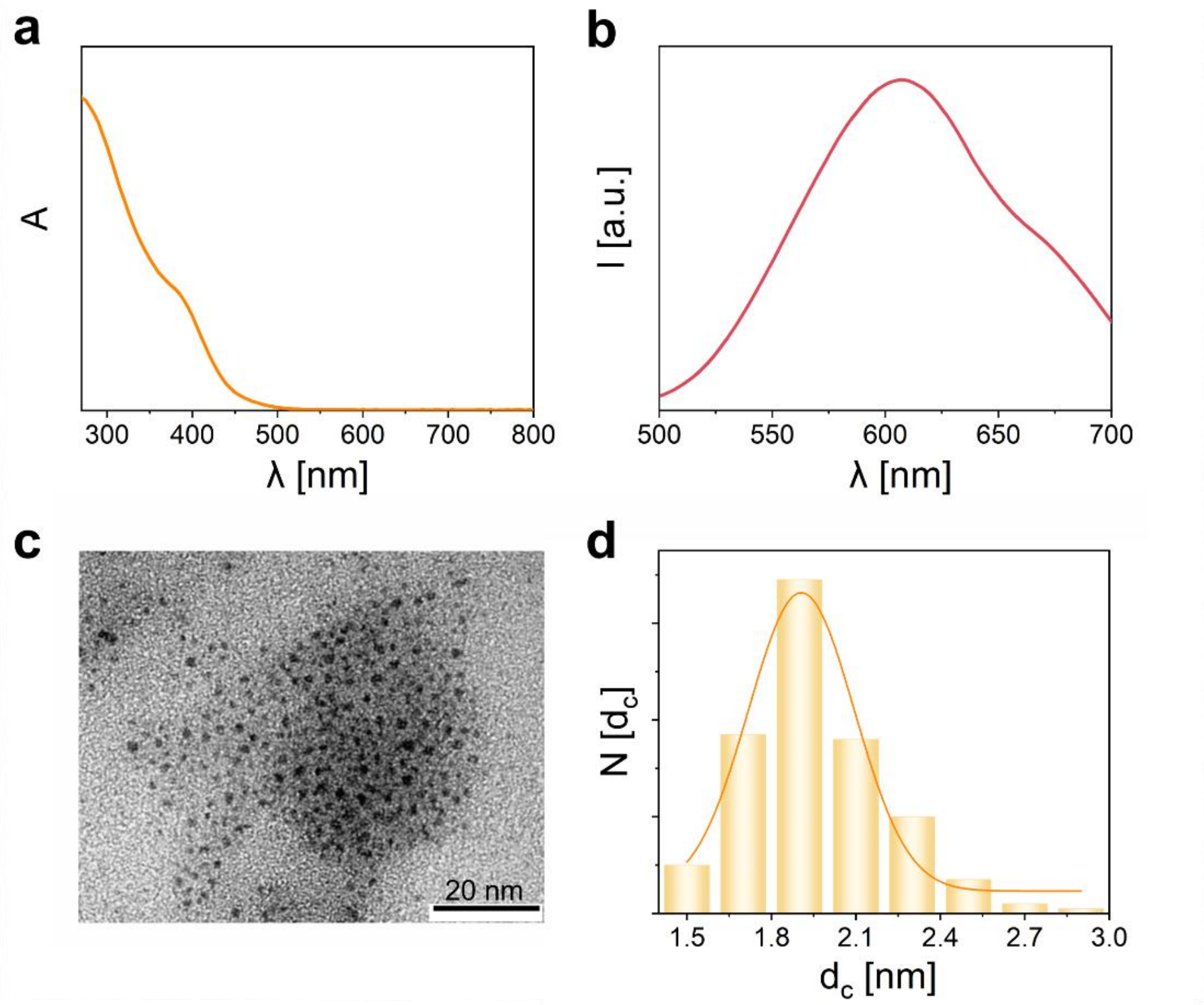


***Fig. S3. Characterization of Au NCs.*** *(a) UV-vis absorption spectrum A(λ) showing a characteristic shoulder peak at ~400 nm (the absence of plasmonic peak at 520 nm confirms the lack of larger nanoparticles which would be present in case of poor size distribution). (b) Photoluminescence spectrum I(λ) with strong emission at 605 nm under 365 nm excitation. (c) TEM image revealing a good size distribution of the NCs. (d) Size distribution histogram N($d_c$) of the core diameter showing an average diameter $d_c$ of 1.9 ± 0.1 nm.*

The Au NCs were synthesized following a previously reported method.[1] As shown in Fig. S3a, the UV-vis absorption spectrum exhibits a distinct shoulder peak at ~400 nm, while no plasmonic absorption band at ~520 nm (typical of larger Au nanoparticles) was observed. This absence confirms the absence of larger nanoparticles and aligns with prior studies,[2] suggesting molecular-like absorption behavior attributable to the discrete electronic states of the Au NCs. Furthermore, the Au NCs demonstrated strong orange fluorescence emission at ~605 nm under 365 nm excitation (Fig. S3b), consistent with earlier reports.[1]

## S2.2 Zeta Potential (ζ) analysis of Au NCs and Au NRs

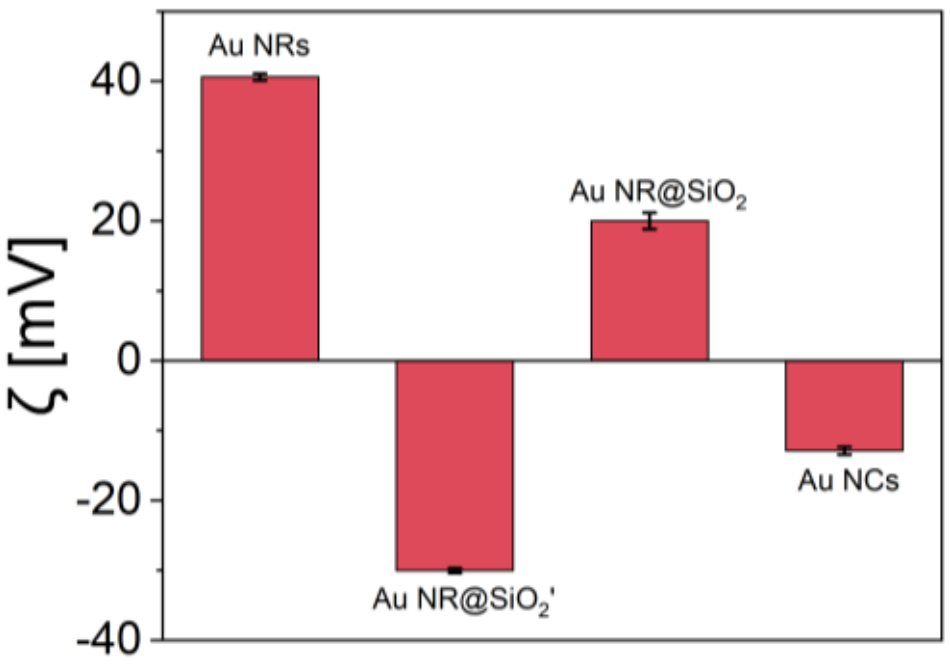


***Fig. S4. Zeta potential (ζ) measurements:*** *Au NRs, Au NR@$SiO_2$' (before functionalization with -$NH_2$), Au NR@$SiO_2$, and Au NCs.*

The as-synthesized CTAB-capped Au NRs displayed a characteristic positive ζ-potential of ζ = +40.6 mV, attributable to the cationic surfactant layer. Following silica coating (Au NR@$SiO_2$'), the ζ-potential reversed to ζ = -30.0 mV, confirming successful $SiO_2$ deposition through the emergence of surface silanol groups. Subsequent functionalization with APTMS yielded Au NRs@$SiO_2$ with a positive potential of ζ = +20 mV, verifying successful amine conjugation. Meanwhile, the Au NCs exhibited a negative potential of ζ = -12.9 mV, consistent with the presence of carboxylate groups from glutathione (GSH) capping ligands. These measurements not only validate the stepwise surface modifications but also confirm sufficient surface charge warranting for the colloidal stability of the nanoparticles.

## S2.3 Optical characterization of nanomaterial-functionalized electrodes

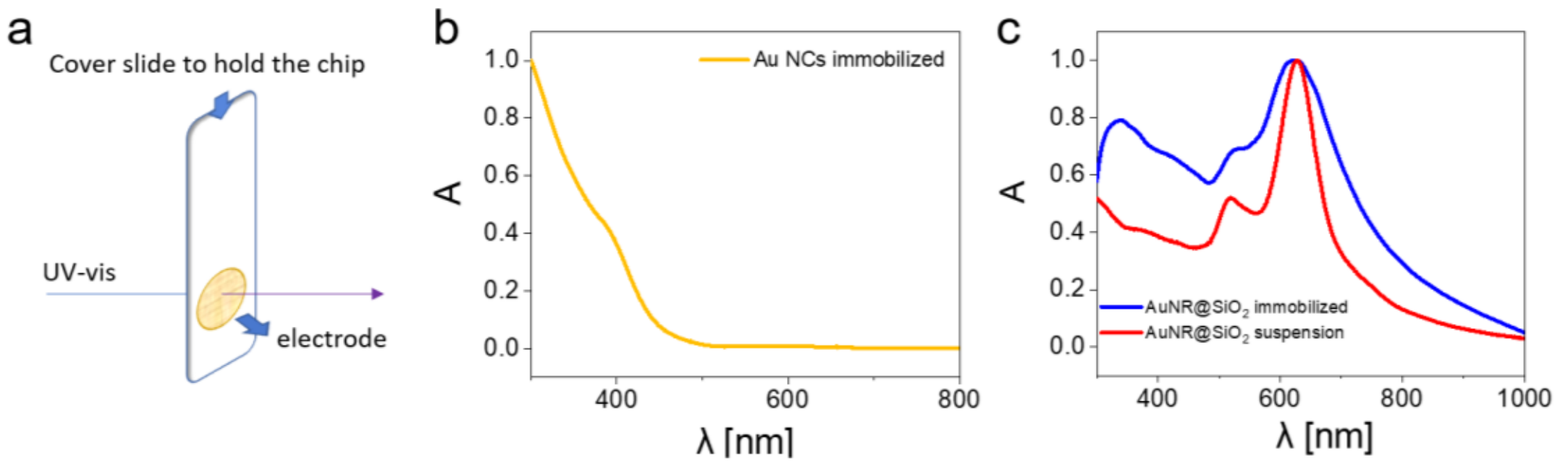


***Fig. S5. Optical characterization of nanomaterial-modified electrodes.*** *(a) Schematic illustration of the absorption measurement setup for Au NRs@*$SiO_2$ *or Au NCs functionalized electrodes. Normalized absorption spectra (A(λ)) of (b) Au NCs and (c) Au NRs@*$SiO_2$ *immobilized on the electrode surface, demonstrating the distinct spectral features of each nanomaterial component in the fabricated PE-PEC platform.*

# S3 FDTD simulation

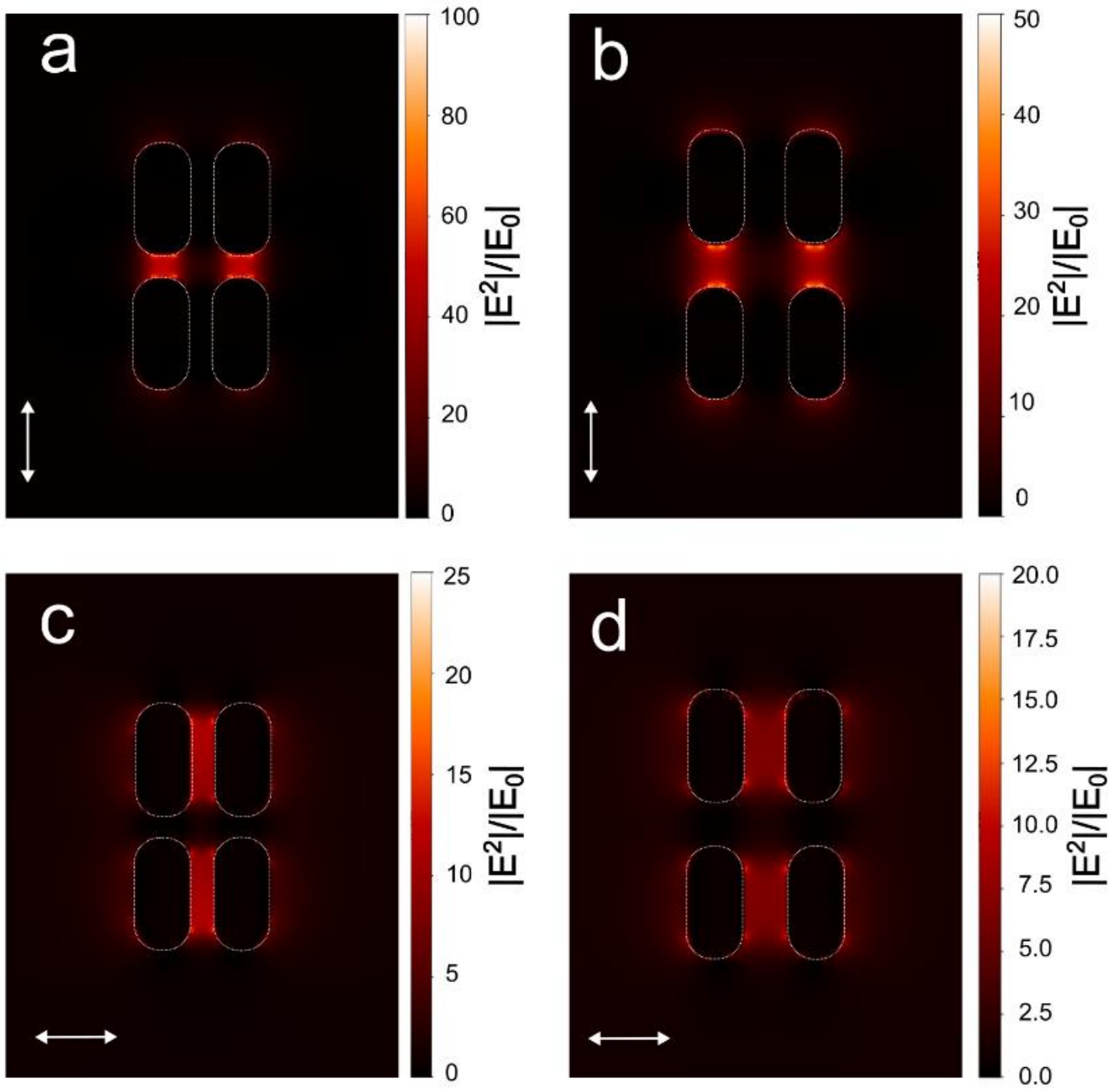


***Fig. S6.*** *FDTD simulation results of normalized EM-field intensity distribution ($|E^2|/|E_0|$) from the XZ plane for* four assembled *Au NR@$SiO_2$ with $SiO_2$ coating of (a,c) $\Delta_s$ = 4 nm and (b,d) $\Delta_s$ = 8 nm in longitudinal (a, b) and transversal direction (c, d), respectively. The Au NR had $l_c$ = 40 nm and $d_c$ = 20 nm in length and width, respectively. The arrows indicate the excitation polarization. The shadow line indicates the position of the single Au NR@$SiO_2$. The colors, from black to yellow, represent the EM field intensity from weak to strong. $|E^2|$ and $|E_0|$ stand for scattered EM field intensity and incident EM-field intensity, respectively.*

# S4 PEC measurement

## S4.1 EIS measurements

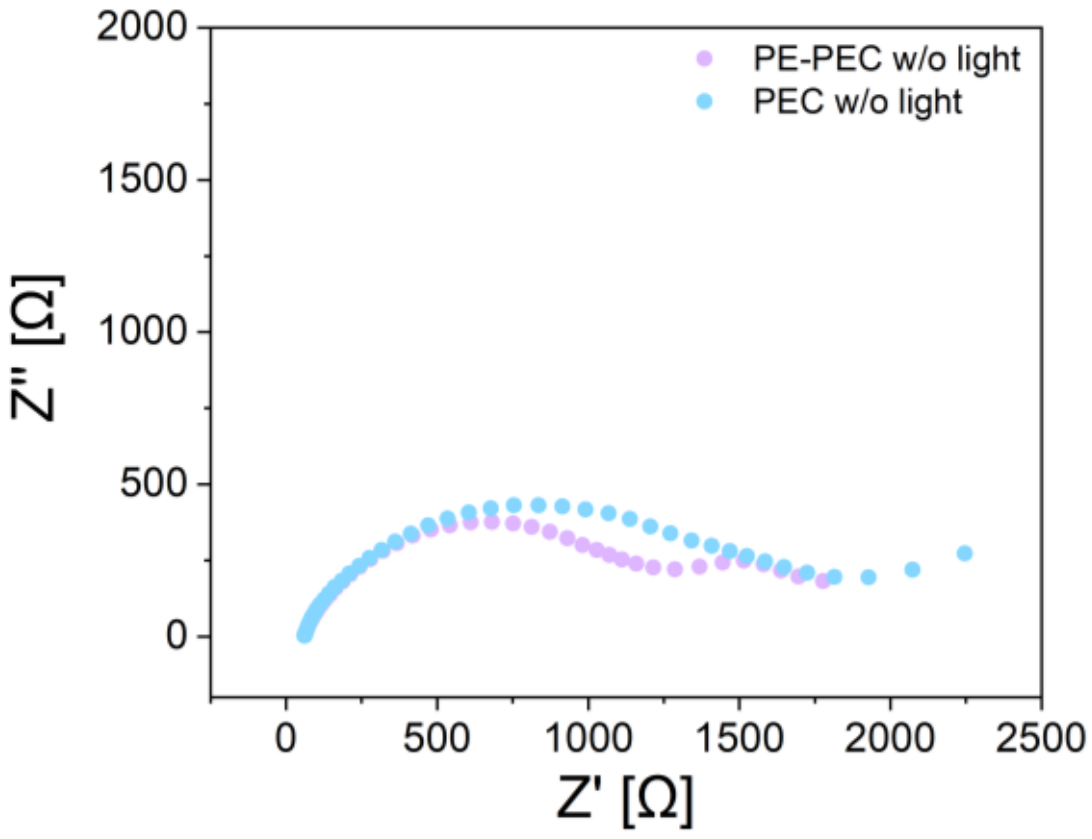


***Fig. S7.*** *EIS characterization of PE-PEC and conventional PEC electrodes without light illumination (w/o light). Measurements were performed at OCP in 10 mM PBS buffer (pH 7.4) containing 5 mM hexacyanoferrate redox couple.*

## S4.2 Characterization of PE-PEC performance

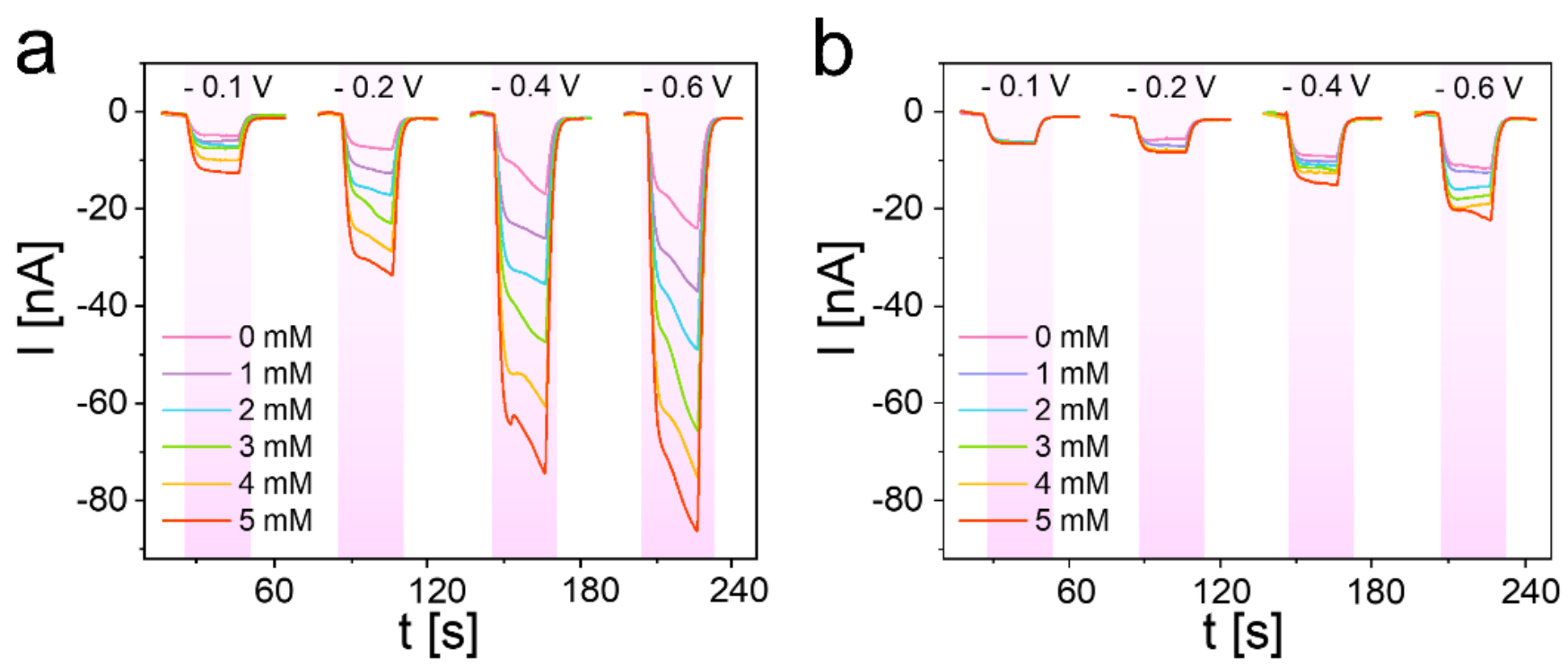


***Fig. S8.*** *Comparative photocurrent response kinetics of (a) plasmon-enhanced (PE-PEC) and (b) conventional PEC electrodes under varying applied potentials (U = -0.1 to -0.6 V vs. Ag/AgCl) with increasing $H_2O_2$ concentrations $c_{H_2O_2}$. Measurements were conducted in 10 mM phosphate-buffered saline (PBS, pH 7.4) under while light illumination.*

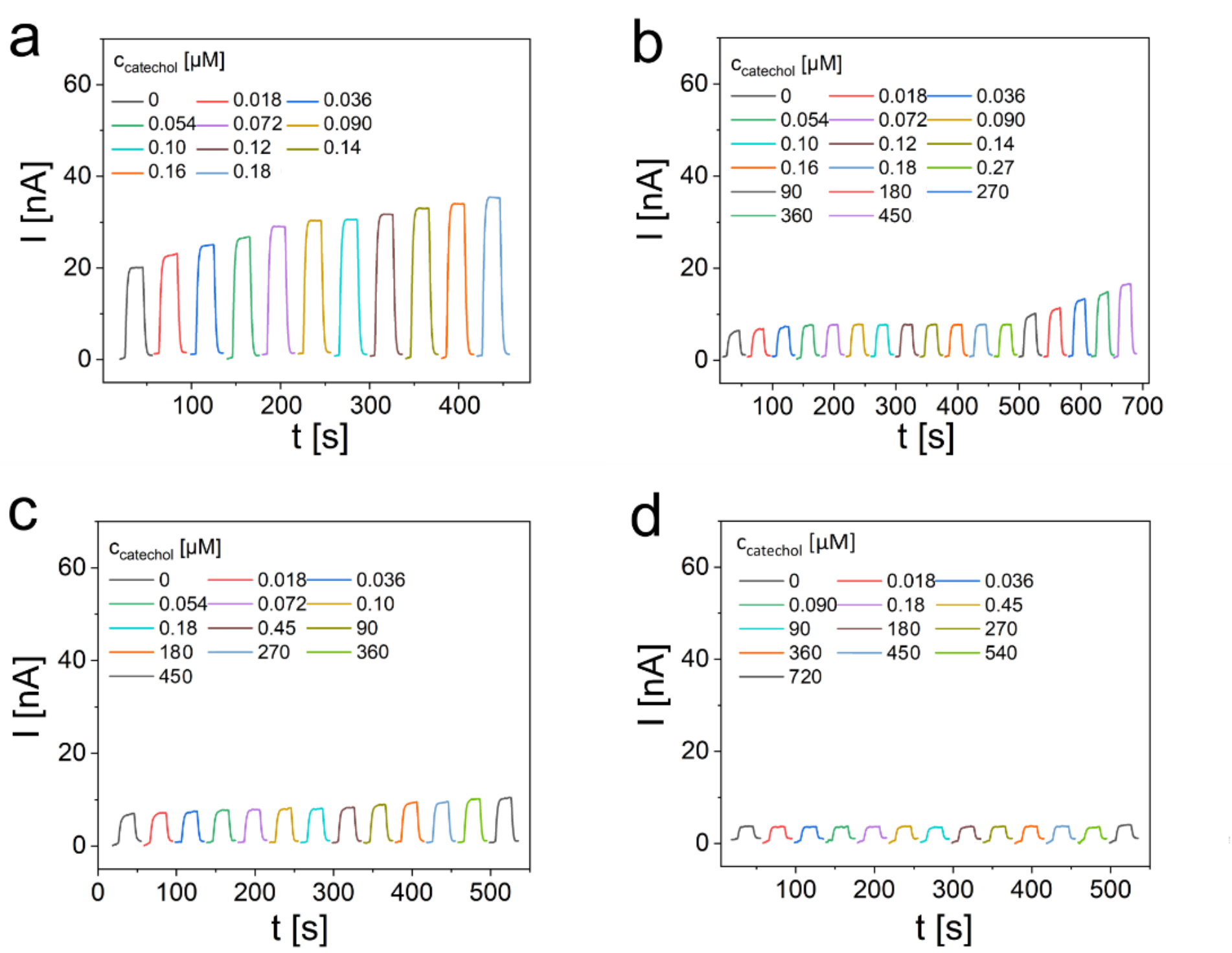


***Fig. S9.*** *Time-resolved current responses of (a) PE-PEC, (b) conventional PEC, (c) AuNR@SiO₂/S-AuE, and (d) bare AuE control under increasing catechol concentrations $c_{catechol}$. Measurements were performed at 0 V vs. Ag/AgCl in 10 mM phosphate-buffered saline (PBS, pH 7.4) under white light illumination.*

## S4.3 Stability and reproducibility of PE-PEC electrodes

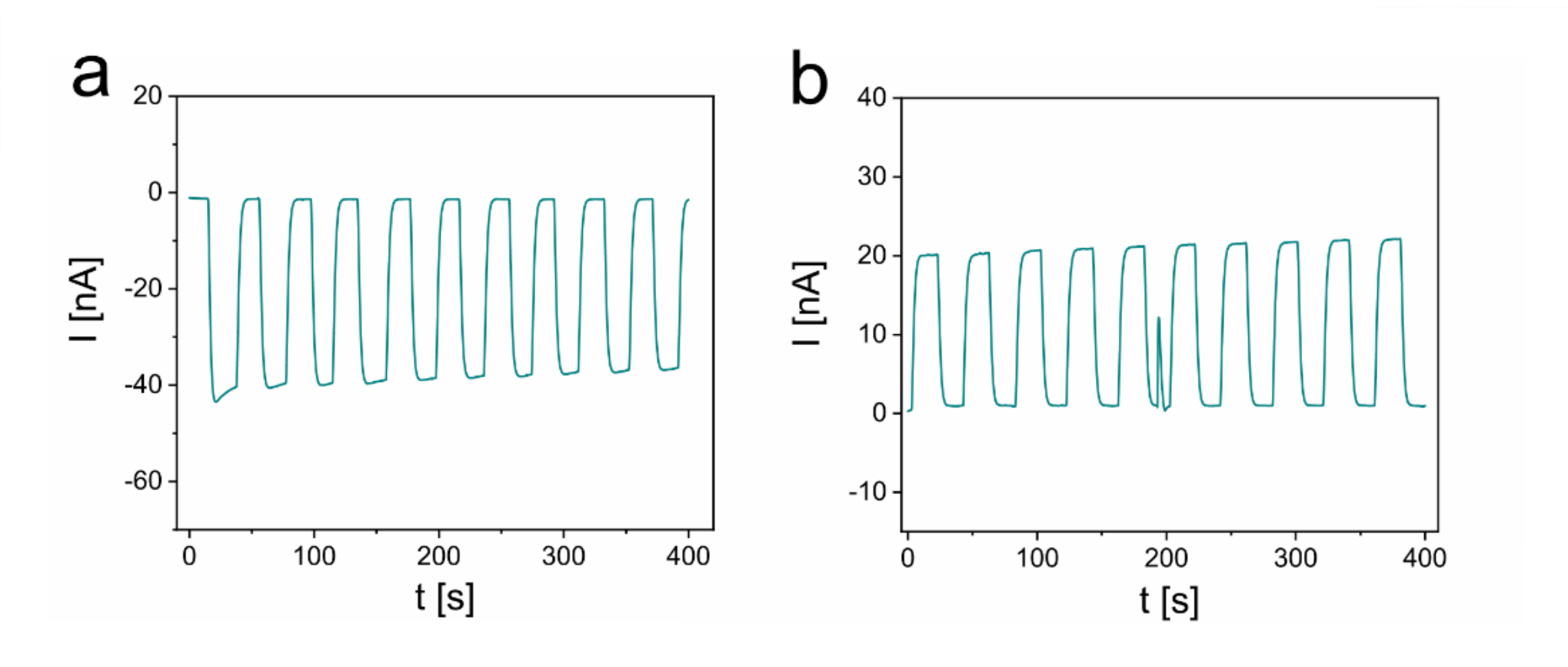


***Fig. S10.*** *Measurements of the photocurrent stability I(t) within 400 s with PE-PEC electrodes under a) 3 mM* $H_2O_2$ *and b) 0.018 µM catechol with a bias U = -0.4 V and 0 V, respectively. The measurements are carried out in 0.01 M PBS pH 7.4 under white light illumination.*

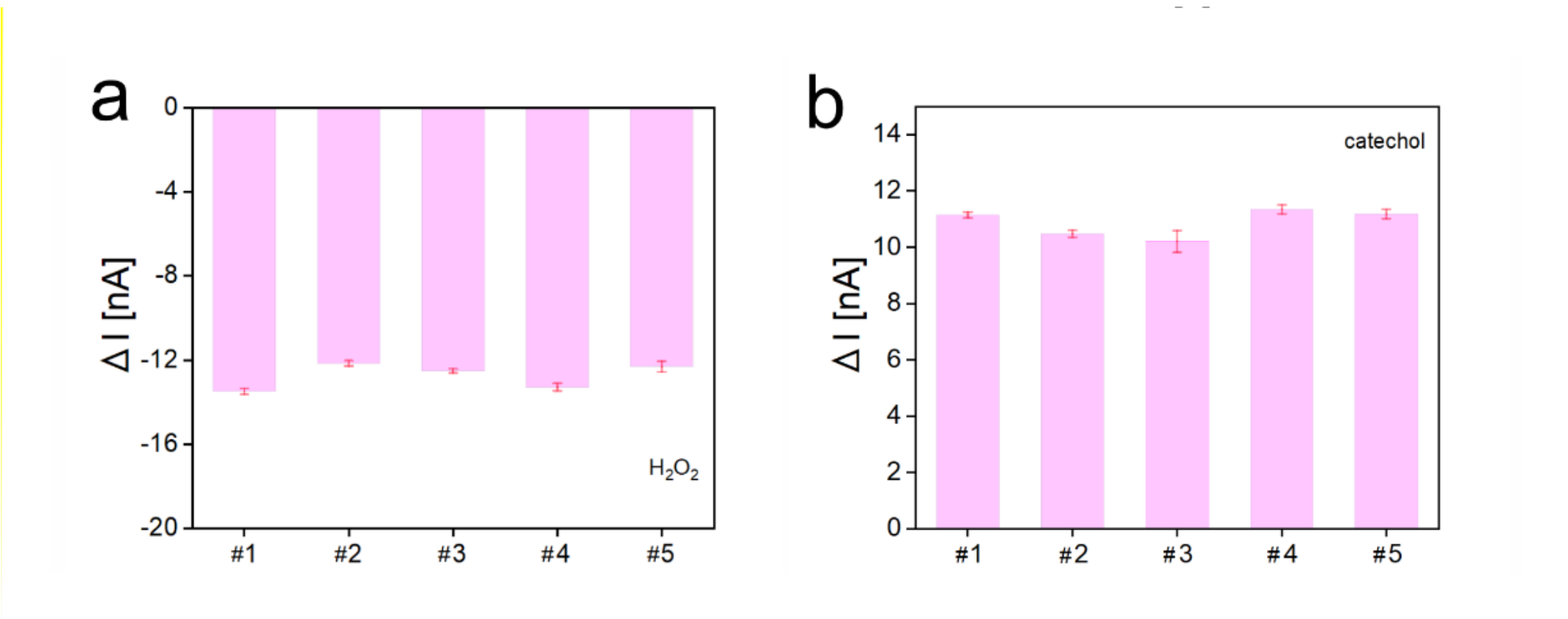


***Fig. S11.*** *Reproducibility of PE-PEC electrodes in (a) 3 mM* $H_2O_2$ *and b) 0.10 µM catechol with U = - 0.2 V and 0 V, respectively.*

***Table S1.*** *Comparison of sensing hydrogen peroxide among different sensing systems (LOD = limit of detection)*

| Sensing Probes | Method | LOD | Reference |
|---|---|---|---|
| 11-Mercaptoundecanoic acid (MUA)-AuNCs | PEC | 35 µM | [3] |
| AuNC/GO hybrid nanocomposites | PEC | 23 µM | [4] |
| CuO | PEC | 60 µM | [5] |
| PbS–$CO_3O_4$ | PEC | 1.2 µM | [6] |
| PE-PEC | PEC | 7.5 µM | This work |

***Table S2.*** *Comparison of sensing catechol among different sensing probes.*

| Sensing Probes | Method | Detection range | LOD | Reference |
|---|---|---|---|---|
| direct green 6 (DG6) decorated carbon paste electrode (CPE) | Electrochemistry (EC) | 5–45 µM | 0.09 µM | [7] |
| AgNPs-Pdop@Gr/glassy carbon electrode (GCE) | EC | 0.5–240 µM | 0.1 µM | [8] |
| Gr/GCE | EC | 1–50 µM | 0.01 µM | [9] |
| $MoO_3/BiVO_4$ | PEC | 0.005–6 mM | 0.41 µM | [10] |
| CNCs/Zn-TPP/GCE | EC | 25–1500 µM | 0.9 µM | [11] |
| $BiPO_4$/BiOI | PEC | 0.06-109 uM | 18 nM | [12] |
| CdS-DNA-pristine graphene | PEC | 0.01–1 uM | 4.9 nM | [13] |
| PE-PEC | PEC | 0.018–0.18 uM | 1 nM | This work |

# S5 References


(1) Luo, Z.; Yuan, X.; Yu, Y.; Zhang, Q.; Leong, D. T.; Lee, J. Y.; Xie, J. From Aggregation-Induced Emission of Au(I)–Thiolate Complexes to Ultrabright Au(0)@Au(I)–Thiolate Core–Shell Nanoclusters. *J. Am. Chem. Soc.* **2012**, *134* (40), 16662–16670. https://doi.org/10.1021/ja306199p.

(2) Zhang, X.-D.; Luo, Z.; Chen, J.; Song, S.; Yuan, X.; Shen, X.; Wang, H.; Sun, Y.; Gao, K.; Zhang, L.; Fan, S.; Leong, D. T.; Guo, M.; Xie, J. Ultrasmall Glutathione-Protected Gold Nanoclusters as Next Generation Radiotherapy Sensitizers with High Tumor Uptake and High Renal Clearance. *Sci. Rep.* **2015**, *5* (1), 8669. https://doi.org/10.1038/srep08669.

(3) Zhang, J.; Tu, L.; Zhao, S.; Liu, G.; Wang, Y.; Wang, Y.; Yue, Z. Fluorescent Gold Nanoclusters Based Photoelectrochemical Sensors for Detection of H2O2 and Glucose. *Biosens. Bioelectron.* **2015**, *67*, 296–302. https://doi.org/10.1016/j.bios.2014.08.037.

(4) Zhao, S.; Li, Z.; Li, Y.; Yu, J.; Liu, G.; Liu, R.; Yue, Z. BSA-AuNCs Based Enhanced Photoelectrochemical Biosensors and Its Potential Use in Multichannel Detections. *J. Photochem. Photobiol. Chem.* **2017**, *342*, 15–24. https://doi.org/10.1016/j.jphotochem.2017.03.034.

(5) Rehosek, M.; Mitoraj, D.; Bledowski, M.; Beranek, R. Photoelectrochemical versus Electrochemical Detection of H2O2 Using an All-Inorganic Sensor Based on Nanostructured Copper Oxide. *Electroanalysis* **2016**, *28* (10), 2327–2334. https://doi.org/10.1002/elan.201600227.

(6) Wang, P.; Cao, L.; Chen, Y.; Wu, Y.; Di, J. Photoelectrochemical Biosensor Based on Co3O4 Nanoenzyme Coupled with PbS Quantum Dots for Hydrogen Peroxide Detection. *ACS Appl. Nano Mater.* **2019**, *2* (4), 2204–2211. https://doi.org/10.1021/acsanm.9b00165.

(7) Chetankumar, K.; Kumara Swamy, B. E.; Sharma, S. C.; Hariprasad, S. A. An Efficient Electrochemical Sensing of Hazardous Catechol and Hydroquinone at Direct Green 6 Decorated Carbon Paste Electrode. *Sci. Rep.* **2021**, *11* (1), 15064. https://doi.org/10.1038/s41598-021-93749-w.

(8) Huang, K.-J.; Wang, L.; Li, J.; Yu, M.; Liu, Y.-M. Electrochemical Sensing of Catechol Using a Glassy Carbon Electrode Modified with a Composite Made from Silver Nanoparticles, Polydopamine, and Graphene. *Microchim. Acta* **2013**, *180* (9), 751–757. https://doi.org/10.1007/s00604-013-0988-5.

(9) Du, H.; Ye, J.; Zhang, J.; Huang, X.; Yu, C. A Voltammetric Sensor Based on Graphene-Modified Electrode for Simultaneous Determination of Catechol and Hydroquinone. *J. Electroanal. Chem.* **2011**, *650* (2), 209–213. https://doi.org/10.1016/j.jelechem.2010.10.002.

(10) Yang, W.; Wang, S.; Ao, J.; Cheng, Q.; Hu, M.; Shu, T. An Unlabeled Dual-Mode Electrochemical and Photoelectrochemical Catechol Sensor Based on MoO3/BiVO4 Heterojunction and Chronoamperometry Method. *Electrochimica Acta* **2025**, *544*, 147668. https://doi.org/10.1016/j.electacta.2025.147668.

(11) Bukhari, S. A. B.; Nasir, H.; Pan, L.; Tasawar, M.; Sohail, M.; Shahbaz, M.; Gul, F.; Sitara, E. Supramolecular Assemblies of Carbon Nanocoils and Tetraphenylporphyrin Derivatives for Sensing of Catechol and Hydroquinone in Aqueous Solution. *Sci. Rep.* **2021**, *11* (1), 5044. https://doi.org/10.1038/s41598-021-84294-7.

(12) Yan, P.; Jiang, D.; Li, H.; Cheng, M.; Xu, L.; Qian, J.; Bao, J.; Xia, J.; Li, H. Exploitation of a Photoelectrochemical Sensing Platform for Catechol Quantitative Determination Using BiPO4 Nanocrystals/BiOI Heterojunction. *Anal. Chim. Acta* **2018**, *1042*, 11–19. https://doi.org/10.1016/j.aca.2018.07.063.

(13) Liu, Y.; Wang, R.; Zhu, Y.; Li, R.; Zhang, J. Photoelectrochemical Sensing of Catechol Based on CdS-DNA-Pristine Graphene Nanocomposite Film. *Sens. Actuators B Chem.* **2015**, *210*, 355–361. https://doi.org/10.1016/j.snb.2014.12.124.
